# Microbial structure and function in infant and juvenile rhesus macaques are primarily affected by age, not vaccination status

**DOI:** 10.1038/s41598-018-34019-0

**Published:** 2018-10-26

**Authors:** Yu Hasegawa, Britni Curtis, Vernon Yutuc, Megan Rulien, Kelly Morrisroe, Kristin Watkins, Clayton Ferrier, Chris English, Laura Hewitson, Carolyn M. Slupsky

**Affiliations:** 1 Department of Food Science and Technology, University of California Davis, Davis, California, USA; 2 Department of Nutrition, University of California Davis, Davis, California USA; 3 Infant Primate Research Laboratory (IPRL), Washington National Primate Research Center, and Center on Human Development and Disability (CHDD), Seattle, Washington USA; 4 The Johnson Center for Child Health and Development, Austin, Texas USA

## Abstract

Although thimerosal, an ethylmercury-based preservative, has been removed from most pediatric vaccines in the United States, some multidose vaccines, such as influenza vaccines, still contain thimerosal. Considering that a growing number of studies indicate involvement of the gut microbiome in infant immune development and vaccine responses, it is important to elucidate the impact of pediatric vaccines, including thimerosal-containing vaccines, on gut microbial structure and function. Here, a non-human primate model was utilized to assess how two vaccine schedules affect the gut microbiome in infants (5–9 days old) and juveniles (77–88 weeks old) through 16S ribosomal RNA sequencing and metabolomics analyses of the fecal samples. Two treatment groups (n = 12/group) followed either the vaccine schedule that was in place during the 1990s (intensive exposure to thimerosal) or an expanded schedule administered in 2008 (prenatal and postnatal exposure to thimerosal mainly via influenza vaccines), and were compared with a control group (n = 16) that received saline injections. The primary impact on gut microbial structure and function was age. Although a few statistically significant impacts of the two common pediatric vaccine schedules were observed when confounding factors were considered, the magnitude of the differences was small, and appeared to be positive with vaccination.

## Introduction

Thimerosal, an ethylmercury (EtHg)-based preservative, has been used in some pediatric vaccines in the United States (US) since the 1930s^1^. In the 1990s, infant exposures of up to 187.5 μg of EtHg by 6 months of age were common in the US^2^, raising concerns about possible developmental effects in children. Since then, a number of studies, both animal and human, have been undertaken. While low concentrations of thimerosal and EtHg found in vaccines was reported to be active against cultured brain cells (reviewed in^3^), data from animal studies was mixed and dependent on the dose of thimerosal used, the mode of administration, as well as methodological differences between studies^4–6^. Both positive and negative effects of thimerosal exposure have been reported in several cohort studies^7–10^. Importantly, studies analyzing the impact of thimerosal on the neurobehavior and brain development using non-human primate models did not show negative outcomes^11,12^. Nonetheless, due to perceived health risks, thimerosal has been removed from most pediatric vaccines in the US, although some multidose vaccines, such as the influenza vaccine and meningococcal vaccine still contain thimerosal^13^.

Curtis *et al*.^11^ studied the impact of US pediatric vaccine schedules, such as that in place during the 1990s and the expanded vaccine schedule recommended in 2008, on multiple neurodevelopmental and cognitive assays using infant rhesus macaques from birth up to 12 months of age. No significant differences were found when comparing the vaccinated groups with the control group that received saline injections^11^. With the same group of primates, Gadad *et al*.^12^ analyzed behavioral outcomes and infant brain development in three brain regions (cerebellum, hippocampus, and amygdala) associated with neuropathology in individuals with autism spectrum disorder (ASD)^12^. No behavioral differences or abnormalities in any of the brain regions were found in the vaccinated groups compared to the control group in terms of neuronal cell morphology, as well as cerebellum expression levels of Purkinje cell-related proteins, (calbindin and glutamic acid decarboxylase) and glial proteins (ionized calcium binding adaptor molecule 1 and glial fibrillary acidic protein).

It is becoming increasingly clear that gut microbiota play strong roles in host biological function, particularly in host immune development via microbe-associated molecular patterns (MAMPs)^14^ and metabolites produced by the gut bacteria^15^. Moreover, a growing number of studies are showing that there is an association between resident gut microbes and vaccine response. For instance, the abundance of *Bifidobacterium* was positively associated with responses to oral and parenteral vaccines in humans^16^. Interestingly, germ-free mice and antibiotic-treated mice show impaired induction of antibodies in the case of vaccination with trivalent inactivated influenza vaccine^17^.

Molecular mechanisms of thimerosal and EtHg transport within the body are not well understood. Human infants injected with thimerosal-containing vaccines (TCVs) showed detectable mercury in stool samples^18^, which suggests that mercury potentially interacts with the gut microbiome. Moreover, it is not clear whether pediatric vaccines would alter the gut microbiota structure and/or function measured through the fecal metabolome. Considering that the gut microbiota plays important roles in host function, it is essential to investigate whether pediatric vaccines might impact the gut microbiota either structurally or functionally. This study utilized a non-human primate model, which allows us to investigate the impact of vaccination on the infant gut microbiota in a system that is closer to humans than rodents, but is still controlled. Here, the impact of TCVs on gut microbial succession in rhesus macaques was studied through analysis of fecal samples obtained from a previous study investigating the effects of pediatric TCVs on neurobehavior and brain development^11,12^.

## Results

### Batch effect on the overall metabolomics and microbiota profiles was minor

**Figure 1:**
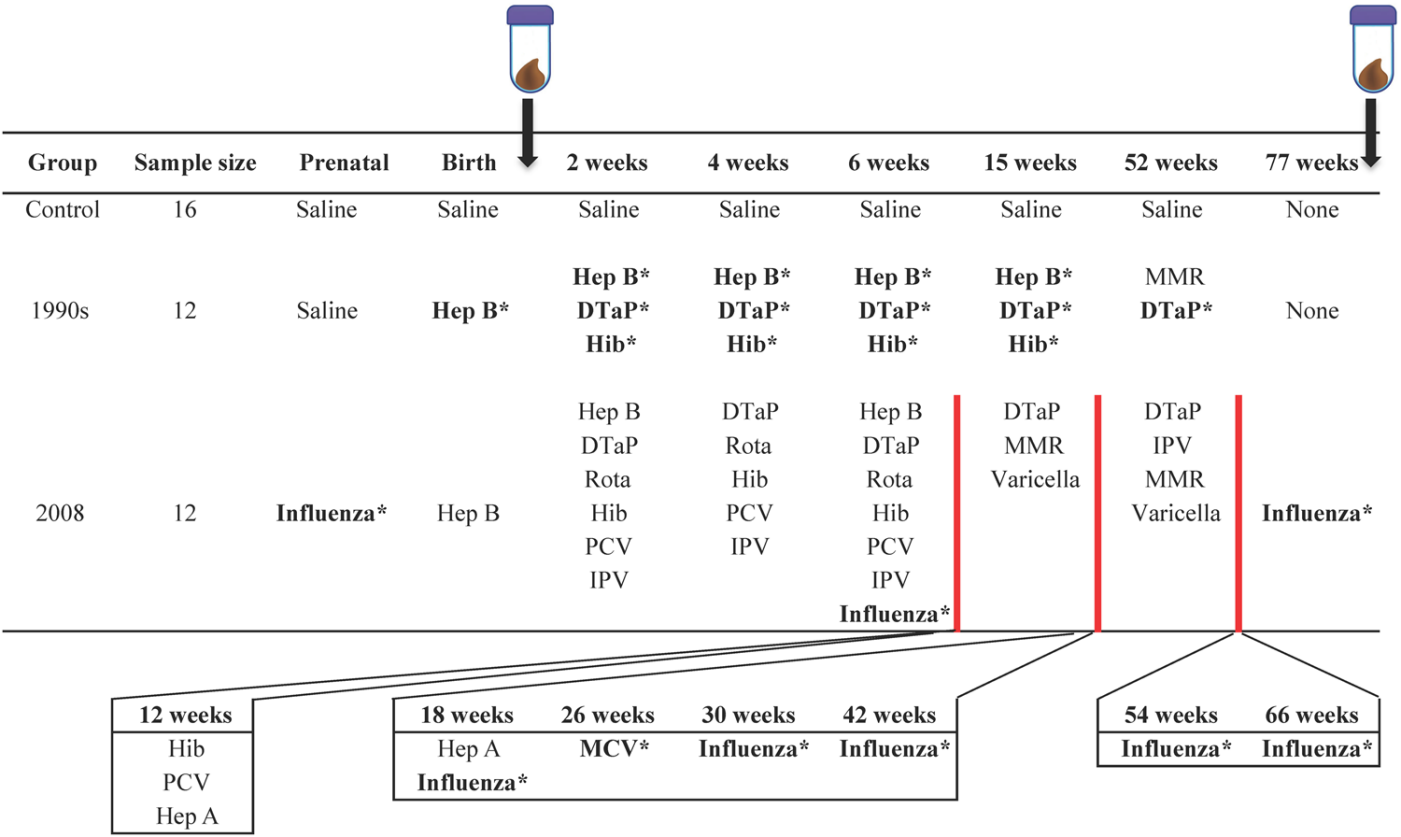
Study groups with the initial sample size and vaccination schedules. Fecal samples were collected at two time points: 5–9 days after the injection of either saline or a birth dose of Hep B vaccination (Infant time point), and when animals were 77–88 weeks old (Juvenile time point). The influenza vaccine was given at 6 weeks and then every 12 weeks to mimic the typical pediatric schedule of yearly vaccination but on an accelerated primate schedule. *Indicates vaccines containing thimerosal. Abbr: Hep B, Hepatitis B vaccine; DTaP, Diphtheria, Tetanus, acellular Pertussis vaccine; Rota, rotavirus vaccine; Hib, Haemophilus influenza B vaccine; MMR, Measles Mumps Rubella vaccine; PCV, pneumococcus vaccine; IPV, inactivated polio vaccine; Varicella, chicken pox vaccine; Hep A, hepatitis A vaccine; MCV, meningococcal vaccine.

The study groups and vaccination schedules are summarized in Fig. 1. Each study group had two or three peer groups of infant macaques born in different years (batches) from 2008 to 2011 (Supplementary Table S1). In order to take the batch effect into account, non-metric multidimensional scaling (NMDS) plots for metabolomics (Supplementary Fig. S1) and both NMDS and alpha-diversity plots for microbiota analyses (Supplementary Figs S2 and S3) were generated. Small R^2^ values, and no significant p-values between batches was observed by permutational multivariate analysis of variance (PERMANOVA) at the Infant time point for either the metabolome (p = 0.081 & R^2^ = 0.14, Supplementary Fig. S1) or microbiota (p = 0.44 & R^2^ = 0.12, Supplementary Fig. S2) data. Although PERMANOVA showed a p-value of 0.001 for both metabolome and microbiota datasets at the Juvenile time point, the R^2^ value was small, suggesting that the batch difference in the centroids of the peer groups was minor (R^2^ = 0.18, Supplementary Fig. S1 and R^2^ = 0.22, Supplementary Fig. S2, respectively). The betadisper test showed that there were no significant differences in the data dispersion among batches at either time point for both metabolome (p = 0.14 at the Infant, p = 0.96 at the Juvenile) and microbiota (p = 0.053 at the Infant, p = 0.087 at the Juvenile time points) datasets. Additionally, no significant differences were observed in alpha diversity among the four batches at either time point (p = 0.53 at the Infant, p = 0.16 at the Juvenile time points, Supplementary Fig. S3).

### Vaccination showed minor impact on microbial function measured in the fecal metabolome

**Figure 2:**
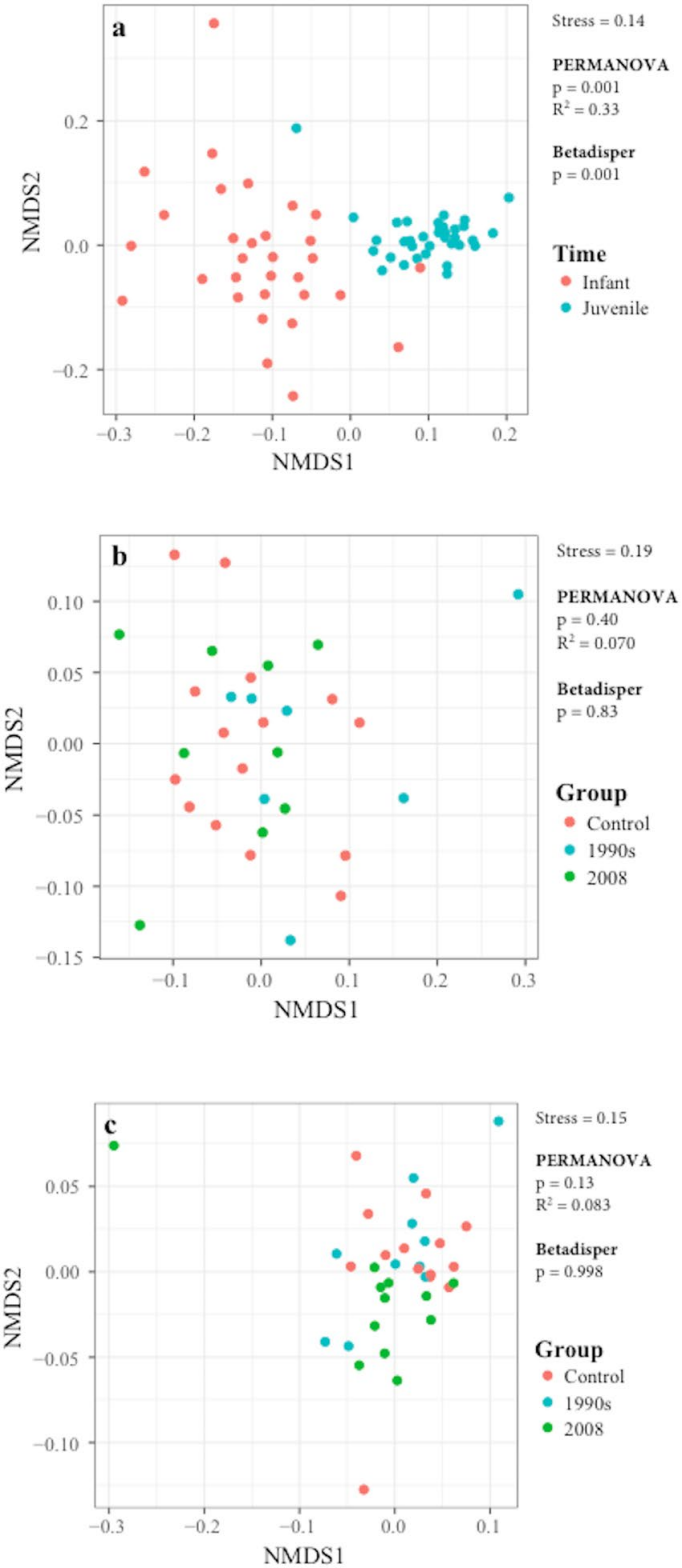
NMDS plots for metabolomics analysis. (**a**) Overall profile of NMDS plot including data from both Infant (red) and Juvenile (blue) time points. NMDS plots for data collected from controls (red), 1990s (blue), and 2008 (green) at the (**a**) Infant and (**c**) Juvenile time points. The stress value, as well as the results from the PERMANOVA and betadisper analyses, are included in each plot.

Differences in microbial functionality as a consequence of vaccination were assessed by NMDS (Fig. 2). The stress value for each plot was acceptably low: 0.14 for the NMDS plot including both time points (Fig. 2a), 0.19 for Infant (Fig. 2b), and 0.15 for Juvenile (Fig. 2c) time points. Although a statistically significant separation was observed between the Infant and Juvenile time points (p = 0.001 by PERMANOVA and p = 0.001 by betadisper, Fig. 2a), no separation among the control and vaccinated groups was observed at either time point by either PERMANOVA (p = 0.40 at the Infant and p = 0.13 at the Juvenile time points, Fig. 2b,c) or the betadisper test (p = 0.83 at the Infant and p = 0.998 at the Juvenile time points, Fig. 2b,c).

Kruskal-Wallis (KW) followed by the pairwise comparison with the Mann-Whitney U test were applied to compare the concentrations of each metabolite among the three groups, and statistical significance was only assumed when the false discovery rate (FDR) adjusted p-values were less than 0.05. Since FDR correction can increase false negative conclusions, the significance was also assessed without FDR correction, and effect sizes were calculated for the post-hoc tests. No significant difference at either time point was observed (Supplementary Table S2). Metabolites with significant differences identified using the KW test prior to the FDR correction were: glucose (p = 0.049) at the Infant time point (higher in the 1990’s vaccination schedule compared with the 2008 schedule and Control), as well as acetate (p = 0.013), 4-aminobutyrate (p = 0.0031), ethanol (p = 0.019), methylamine (p = 0.023), propionate (p = 0.018), and valerate (p = 0.043) at the Juvenile time point (all higher in the 2008 vaccination schedule compared with the 1990’s schedule and Control) (Supplementary Fig. S4 and Table S2). Although most of these metabolites showed insignificant p-values in post-hoc tests, two metabolites showed significant p-values with large effect sizes: acetate (p = 0.015 & r = 0.60) and 4-aminobutyrate (p = 0.0052 & r = 0.67), which were both higher in the fecal metabolome of infants receiving the 2008 vaccination schedule compared with the 1990’s schedule. In order to address the possibility that our small sample size made it difficult to detect significant statistical differences after FDR correction, the statistical power and 95% confidence intervals (CI) were calculated (Supplementary Table S2). Utilizing bootstrapping, the statistical power was found to be medium to high, and the 95% CI showed similar trends as indicated above.

Non-parametric analysis of covariance (ANCOVA) with FDR correction, as well as NMDS plots revealed that there were no significant differences among control and vaccinated groups as a function of potential confounding factors, such as age (Supplementary Table S1) for both time points, and drug administration (Supplementary Table S3) for the Juvenile time point, which are summarized in Supplementary Table S4 and Supplementary Fig. S5. Also, exclusion of animals with any medication resulted in no significant differences in the centroids among the three groups according to the NMDS plots (Supplementary Fig. S6) and KW test after FDR correction (Supplementary Table S5). The batch effect was further evaluated using non-parametric ANCOVA, which revealed no metabolites with significant differences among the three groups after FDR correction (Supplementary Table S6).

### Vaccination showed minor impact on the structure of the gut microbiota

**Figure 3:**
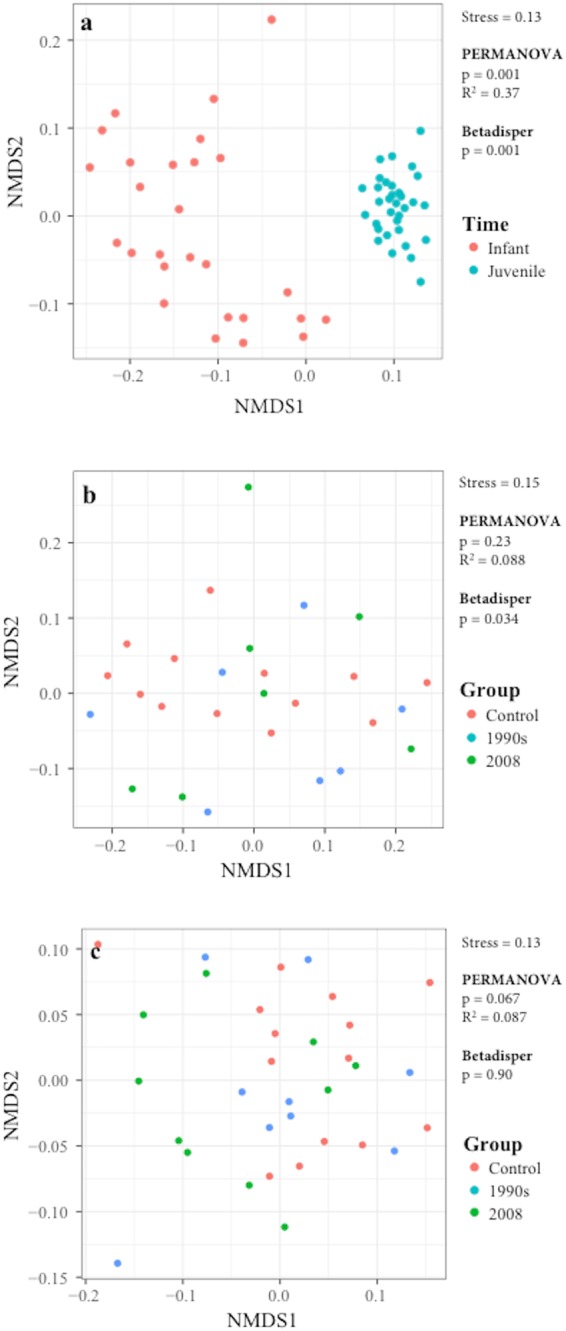
NMDS plots for microbiota analysis based on weighted UniFrac distance. (**a**) Overall NMDS plot including data from both at the Infant (red) and Juvenile (blue) time points. NMDS plots for data collected from controls (red), 1990s (blue), and 2008 (green) groups at the (**b**) Infant and (**c**) Juvenile time points. The stress value, as well as the results from PERMANOVA and betadisper analyses, are incuded in each plot.

**Figure 4:**
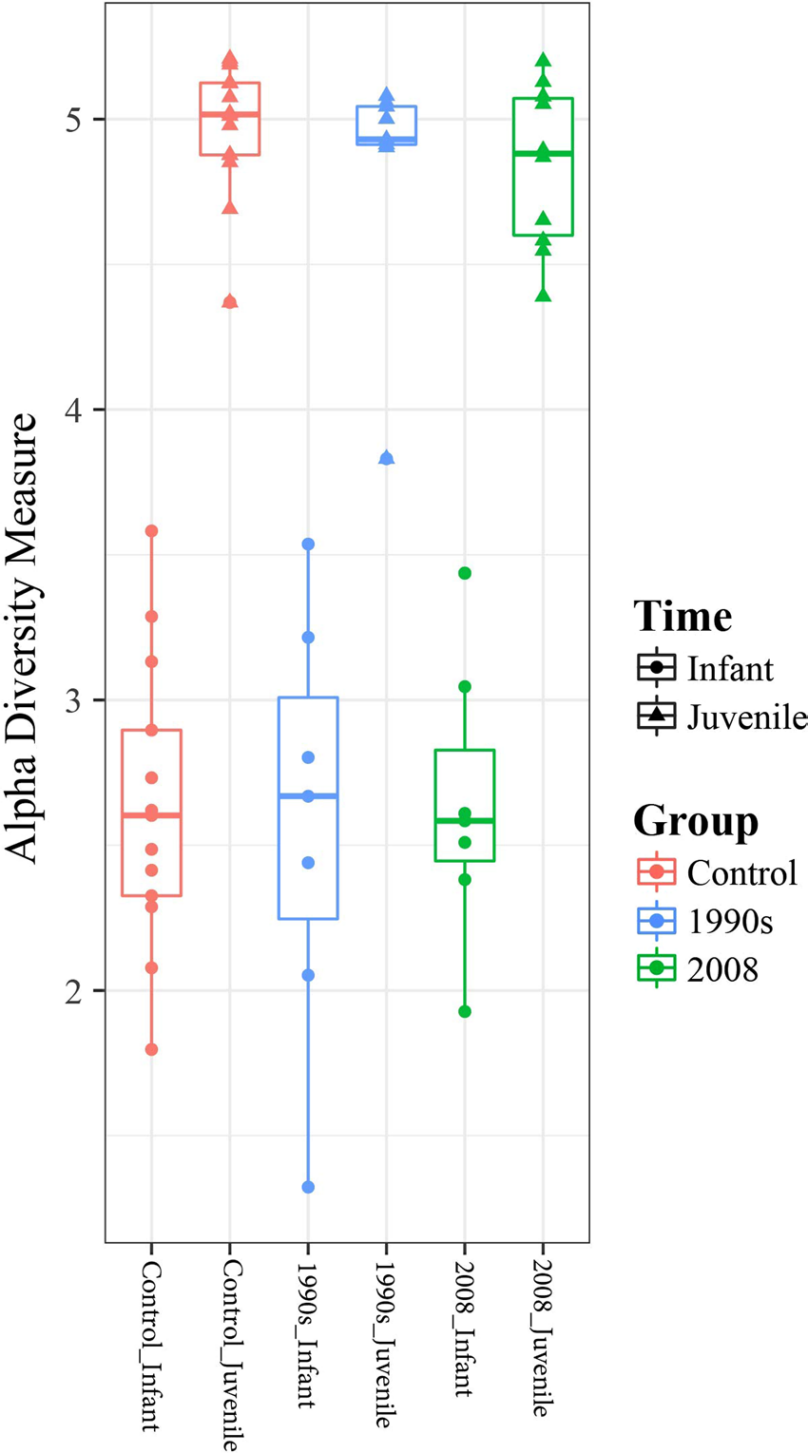
Box plot showing the alpha diversity values for controls (red), 1990s (blue), and 2008 (green) groups based on the Shannon method. Circles represent data at the Infant time point, and triangles represent data at the Juvenile time point. The middle line in the box represents median of the data, and the box represents lower and higher quantiles. The edges of whiskers represent the lowest and highest values of the dataset.

Beta diversity in the gut microbiota was visualized using NMDS plots (Fig. 3), which revealed low stress values: 0.13 for the NMDS plot including both time points (Fig. 3a), 0.15 for Infant (Fig. 3b), and 0.13 for Juvenile (Fig. 3c) time points. The largest separation was observed between the two sample collection time points (Fig. 3a). The control and vaccinated groups did not show statistically significant separation in the distribution on the NMDS plots at either the Infant (Fig. 3b) or the Juvenile (Fig. 3c) time points. The betadisper test suggested that there was a significant difference in the homogeneity of group dispersion between the control and the 2008 group at the Infant time point (p = 0.034 for betadisper test, and p = 0.033 for TukeyHSD test comparing Control and 2008). However, at the Infant time point, PERMANOVA revealed a p-value of 0.23 and an R^2^ value of 0.088, meaning that only 8.8% of the variation could be explained by the Group, which suggests that the centroids of the three groups were not significantly different (Fig. 3b). The richness of the microbiota was assessed as alpha diversity using the Shannon method (Fig. 4). Alpha diversity was not significantly different between the three groups at either time point (p = 0.99 and p = 0.54 at the Infant and at the Juvenile time points, respectively). Other statistical methods to assess alpha diversity were also tested and no statistically significant differences among the control and vaccinated groups were observed at either time point (Supplementary Table S7 and Supplementary Fig. S7).

Relative abundance at the genus level was assessed at each time point (Supplementary Table S8). At the Infant time point, *Bifidobacterium* dominated all groups, although its relative abundance was not significantly different among the groups (an average of 30.45% for control, 32.11% for 1990s, and 20.10% for 2008). At the Juvenile time point, either *Lactobacillus* or *Prevotella* dominated all groups (an average of 7.27% and 7.72% for control, 14.27% and 7.41% for 1990s, and 14.04% and 9.34% for 2008, respectively). DESeq2 was used to identify bacteria with significant differences in abundance among the three groups, and p-values were generated by the likelihood ratio test. Bacterial abundance with an estimated fold difference of higher than 4 or lower than ¼ together with a base mean (the average of the normalized OTU counts of all samples) higher than 30 and adjusted p-value of less than 0.05 were considered to be significantly different. As a result, with medium to high statistical power, no bacteria showed significant p-values with FDR correction at the genus (Supplementary Table S9), family (Supplementary Table S10), or order levels (Supplementary Table S11). Prior to FDR correction, some bacteria showed p-values lower than 0.05: at the genus level, *Aggregatibacter* and *Enterococcus* at the Infant, and *Mogibacterium* at the Juvenile time point; at the family level, *Bacteroidaceae*, *Enterobacteriaceae*, and *Enterococcaceae* at the Infant, and *Erysipelotrichaceae* at the Juvenile time point; at the order level, *Enterobacteriales* at the Infant, and *RD32* at the Juvenile time point (Supplementary Table S9–11 and Supplementary Fig. S8).

The same statistics were applied to include potential confounding factors, including age for both time points, drug administration for the Juvenile time point (Supplementary Fig. S5 and Supplementary Table S12), and the batch effect (Supplementary Table S13). Upon correcting for age at the Infant time point, one genus of bacteria, *Enterococcus*, showed an adjusted p-value of less than 0.05 together with a large effect size, which may be due to the two outliers that showed very high OTU counts compared to the rest of the animals in the study group (Supplementary Fig. S8). NMDS plots generated with animals without any medications showed significant differences in the centroids of the three groups at the Juvenile time point (Supplementary Fig. S9) although the R^2^ value indicates that relatively small portion of the variation (27%) could be explained by the Group. Furthermore, the likelihood ratio test in DESeq2 was applied on animals that did not take any medications, and one genus of bacteria, *Lachnospira*, was determined to be significantly different between the 1990s and 2008 groups at the Juvenile time point with an adjusted p-value of 0.0058. Its bacterial abundance in the 1990s group showed an estimated 0.17 fold lower relative abundance than the 2008 group (Supplementary Table S14, Supplementary Fig. 10).

## Discussion

In a number of countries around the world including the US, infants are vaccinated soon after birth^13^, as well as indirectly exposed *in utero* through a pre-natal influenza vaccination. In the 1990s, a number of pediatric vaccines given to infants contained thimerosal, a mercury-based preservative, which led some parent and advocacy groups to question the safety of vaccines because the cumulative thimerosal exposure in some US children was 187.5 μg EtHg by 6 months of age^2^. While thimerosal has generally been removed from most pediatric vaccines, it is still common for US mothers to receive seasonal influenza vaccines during pregnancy that contain thimerosal. The infant gut microbiota is involved in the development of the host immune system as well as in neurobehavioral and brain development^19,20^. In order to further investigate the safety of TCVs, this study examined the influence of TCVs on the gut microbiota and the fecal metabolome in a non-human primate model.

Infant macaques vaccinated following the pediatric vaccination schedule recommended in the 1990s received a thimerosal-containing hepatitis B (Hep B) vaccine at birth. For those animals receiving the recommended 2008 pediatric vaccine schedule, the first exposure to thimerosal was *in utero* as their mothers received a thimerosal-containing influenza vaccine during the last month of pregnancy. A thimerosal-free Hep B vaccine was administered at birth (since thimerosal had been removed from most pediatric vaccines by 2003). By comparing the results from macaques receiving vaccines according to the recommended 1990s and 2008 schedules with a control group (receiving only saline injections), it is possible to observe how thimerosal exposure through either pre-natal or post-natal routes could impact the gut microbiota in infant and juvenile macaques.

Once a TCV is administered, it is immediately dispersed in the blood stream. Thimerosal likely goes to the liver where it is broken down into EtHg and thiosalicylic acid, and possibly from EtHg to inorganic Hg through enzymatic activity^21,22^. Feces from human infants who received a series of TCVs were shown to have a detectable concentration of Hg that was mainly inorganic^18^. Inorganic Hg is primarily excreted from the body through the feces. As the terminal half-life of blood Hg after thimerosal injection in infant macaques is approximately 8.6 days^23^, gut microbiota observed in the macaques receiving the 1990s vaccination schedule at the Infant time point may reflect the impact of exposure to both EtHg and inorganic Hg. However, neither the structure nor metabolic function of the gut microbiota was significantly different between animals in the 1990s and control groups at the Infant time point. These results suggest that the single dose of thimerosal at birth from vaccination with the Hep B vaccine did not have a significant impact on the gut microbiota.

There is limited understanding of whether injected thimerosal or its metabolic products can be transferred through the placenta to enter the uterus. Although the placenta is impermeable to inorganic Hg^24^, organic mercury such as methylmercury (MeHg) crosses the placenta and can accumulate within the fetus possibly disturbing fetal brain development^25,26^. It is therefore conceivable that thimerosal itself, or its metabolite EtHg, could pass through the placenta, impact the fetal gastrointestinal tract, and thus impact the establishment of gut microbiota in the neonate before EtHg is further broken down into inorganic Hg within the maternal organs. However, both microbiota and metabolome analyses did not show significant differences between the group receiving the 2008 vaccine schedule (which included a prenatal influenza vaccine) and the control group at the Infant time point, suggesting that thimerosal injected via a TCV during the last month of pregnancy in rhesus macaques, does not significantly affect the neonatal gut microbiota.

Assessing samples collected at the Juvenile time point provided information regarding the influence of thimerosal over the entire vaccination schedule. While the total thimerosal exposure for the 1990s and 2008 animals was fairly similar (41.58 μg EtHg versus 31.68 μg EtHg, respectively), the vaccination schedules were very different. Animals in 1990s group were exposed to thimerosal more intensely consisting of 14 vaccines over the course of the study, providing 37.62 μg EtHg by 15 weeks of age. Those in the 2008 group, on the other hand, followed an expanded vaccine schedule consisting of 37 vaccines over the course of the study, providing 3.96 μg EtHg by 15 weeks of age. Animals in this group received thimerosal primarily from influenza vaccines starting at 6 weeks of age and continuing every 12 weeks until the end of the study. A single meningococcal vaccine administered at 26 weeks of age also contained thimerosal. Our results suggest that the impact of the two vaccination schedules on the structure and function of the gut microbes was not significantly different from controls as assessed through metabolomics and microbiota analysis of fecal material.

The only significant difference in the microbiota noted was between the Infant and Juvenile samples when the three study groups were combined, which suggests that the biggest influence on microbial function in both infant and juvenile macaques is age as has been reported previously^27,28^. Alpha diversity was also significantly different between Infant and Juvenile samples but was not affected by vaccination status. The most common genus in the Infant samples across all groups was *Bifidobacterium* and *Blautia*, whereas in Juvenile samples, *Lactobacillus*, *Prevotella* and *Ruminococcus* were the most abundant genera. Using fecal microbial profiling, formula-fed rhesus infants have previously been reported to have low levels of *Ruminococcus* and *Lactobacillus* genera at birth, with these levels increasing after 3 months of age^29^, suggesting that the levels of bacteria from these two genera increase by 3 months of age and are still abundant at 18 months of age. Furthermore, the concentrations of some metabolites (specifically, acetate and propionate which are short chain fatty acids primarily produced by bacterial fermentation of non-digestible carbohydrates as well as amino acids^30^) were consistently higher in 2008 compared to the other two groups at the Juvenile time point, although there were no statistically significant differences in the bacterial relative abundance between 2008 and the other two groups. Furthermore, 4-aminobutyrate was found to be higher in the 2008 group compared to the other two groups, although the KW test with FDR correction showed an insignificant p-value. 4-aminobutyrate is also known as γ-aminobuytric acid (GABA), which acts as an excitatory transmitter during the early stage of life and is involved in brain development^31^. These changes appear to be beneficial considering the properties of the metabolites affected. When bacterial abundance was compared between animals without any medication, the abundance of *Lachnospira* was significantly higher in the 2008 cohort compared to 1990s, and higher than Control at the Juvenile time point, which may explain the increase in the concentrations of acetate and ethanol. *Lachnospira* has been reported to exist in the gastrointestinal tract of humans and pigs, as well as the rumen of cattle. Species from this genus metabolizes polygalacturonic acid, the main component of pectin, to produce acetate and formate as major, and ethanol as minor end products^32^.

In this study, both p-values, which represent the probability that the null hypothesis is accepted or rejected, and effect sizes were reported in order to provide a more complete picture of the significance of an observation. A strong effect size with an insignificant p-value suggests that with additional subjects, significance might be observed. Therefore, these trends in the data were reported since they could have biological meaning. There are a number of limitations in this study. The sample size is small and as such, the findings should be interpreted with caution. Furthermore, the sample size of the infants born by Caesarean section (C section) versus vaginal delivery was very small, which was not robust enough to assess its impact on the metabolome and microbiota, although the mode of delivery did not appear to impact clustering. Due to the size of this study (76 animals in the original study), infants were born over 4 breeding seasons, which could impact environmental exposures. However, batch effects examining year of birth as a confounding variable were not significant. Finally, some animals were administered various drugs during the study including antibiotics. This did not impact the Infant samples but, like any housed primate colony, giardia and cryptosporidium outbreaks occur periodically. When this occurred, all animals in the same room as the infected animal were treated prophylactically resulting in multiple potential drug exposures. This is the first study to our knowledge that has measured the impact of vaccination, especially TCVs, on macaque infant gut microbial succession through metabolomic and microbiota analysis of infants soon after birth and of juveniles at 18 months of age. In this controlled animal study, the primary impact on the gut microbiome was age. We noted a few statistically significant differences on the gut microbiome structure between vaccinated and non-vaccinated groups when only animals without any medication were analyzed, but these differences were small, and appeared to be positive changes.

## Methods

### Animals

Protocols were approved by the University of Washington Institutional Animal Care and Use Committee and were conducted in accordance with the Animal Welfare Act and the *Guide for Care* and *Use of Laboratory Animals* (National Research Council 2011). Rhesus macaques (*Macaca mulatta*) were bred through natural mating at the California National Primate Research Center (CNPRC) in Davis, California and selected for transport to the Washington National Primate Research Center (WaNPRC) Infant Primate Research Laboratory (IPRL) after ultrasound confirmation of a male fetus. Pregnant dams were monitored by infrared cameras until delivery. If labor was delayed more than 7 days past a dam’s due date or if any other medical concerns were noted on clinical observation, a C section was performed. In this analysis, 6 out of 40 infants were born via C section: one control, two 1990s, and three 2008 (Supplementary Table S1).

Each infant received standard neonatal care and was raised individually in their home cage in the same rearing room as the other members of their peer group following well established protocols^11,33^. At approximately 12 months of age, each peer group of 4 animals were group-housed with daily access to the playroom. Infants received standard infant formula (Enfamil Premium with iron, Enfamil, Glenview IL). Animal biscuits (containing approximately 15% wheat gluten; Purina Mills, St. Louis, MO) were introduced during the first month but rarely ingested until about 3 months of age. At approximately 4 months of age, infants were weaned off formula. Fresh fruit and vegetables were provided daily as standard enrichment. Water was available *ad libitum*.

### Medications

No medications were administered to infants prior to the first fecal collection. A number of animals across all three groups received various medications during the course of this study. The clinical indication for treatment, medication administered, length of treatment, and the number of days between the completion of treatment and the collection of the Juvenile fecal sample are summarized in Supplementary Table S3. Two animals (one control and one 1990s) tested positive for giardia. Sixteen additional animals that were in the nursery during a giardia outbreak received prophylactic treatment. Nine animals (four control, two 1990s and three 2008) displayed dermal or joint inflammation and received additional antibiotic treatment. Finally, three animals (two control and one 2008) received a probiotic (*Lactobacillus*) to aid with diarrhea. The number of days between completing treatment with antibiotics and the collection of the juvenile stool sample was at least 60 days except for one control.

### Vaccination schedules

In this study, 40 male infant macaques were analyzed in 3 groups: control (animals received saline injections only in place of all vaccines, n = 16), 1990s (animals were vaccinated according to the recommended pediatric vaccination schedule in the 1990s, n = 12), and 2008 (animals were vaccinated according to the recommended pediatric vaccination schedule group in 2008, which remains similar to the schedule of today, n = 12). The study groups and vaccination schedules are summarized in Fig. 1. In order to adjust the timing of vaccination to accommodate the projected 4:1 developmental trajectory of infant primates compared with humans^11^, we used a truncated vaccination schedule accelerated approximately four-fold. The vaccine manufacturer, thimerosal content and route of administration are shown in Supplementary Table S15.

### Vaccine Dosing

To recreate the TCVs, thimerosal-free vaccines were purchased, pooled, and then dosed with a thimerosal stock solution to produce the desired concentrations. TCVs were produced by the Environmental Research Training Lab at the University of Kentucky, and periodically tested throughout the study using an independent testing laboratory (Quicksilver Scientific, Lafayette, CO) to confirm thimerosal concentration. Thimerosal concentrations in each vaccine were standardized to maximize possible exposure while still maintaining an appropriate clinical exposure level for infant macaques.

### Fecal collection

Fecal samples were collected 5–9 days after the birth dose of vaccine (Infant time point) and at necropsy (Juvenile time point) when the macaques had received all the scheduled vaccinations and were approximately 80 weeks old. For the Infant sample, fresh fecal samples were collected opportunistically during infant handling or directly from the white cloth diaper in each isolette. For the Juvenile sample, fecal samples were collected directly from the colon during necropsy. All fecal samples were frozen and stored at −80 °C until analysis. Three Infant samples: one control and two 1990s were not available for analysis (Supplementary Table S1).

### ^1^H NMR spectroscopy sample preparation

Fecal samples were removed from the −80 °C freezer and allowed to thaw on ice. Approximately 250 mg of feces was weighed and mixed with 1,500 μL of ice-cold PBS in to extract metabolites. The supernatant was filtered with 0.22 μm Millex GP PES membrane syringe filter (Millipore, Billerica, MA) followed by filtration with Amicon Ultra-0.5 mL centrifugal filter (3k MW cutoff, Millopore, Billerica, MA) in order to remove proteins and lipids. 23 μL of 5 mM 3-trimethylsilyl-1-propanesulfonic acid-d_6_ (DSS-d6) was added as internal standard to 207 μL of the filtrate. One sample of 1990s at the Infant time point was lost during the metabolite extraction procedure. Samples were stored at 4 °C overnight. pH was adjusted to 6.8 +/− 0.1 by adding a small volume of either 1 N NaOH or 1 N HCl in order to minimize peak shift driven by pH. 180 μL of sample was transferred to a 3 mm Bruker NMR tubes (Bruker, Billerica, MA). Samples were kept at 4 °C until NMR acquisition.

### NMR data acquisition and spectral processing

All ^1^H NMR spectra were acquired using the noseypr1d pulse sequence on an Advance 600 MHz spectrometer at 25 °C (Bruker, Billerica, MA). For all NMR spectra, Fourier transformation, phase, and baseline correction were manually applied by using Processor in Chenomx NMRSuite (version 8.1, Chenomx Inc., Edmonton, Canada). Concentrations of metabolites (μM) were determined in Profiler by comparison of the area of the metabolite to that of an internal standard (DSS-d6) as the reference, and converted to units of nmol/g dry weight. The dry weight of the fecal sample was calculated by determining the amount of water after drying a specific weight of wet fecal matter using a miVac (Genevac ltd., Ipswich, England).

### DNA extraction, purification, library construction and sequencing

The MoBio PowerLyzer PowerSoil DNA isolation kit (MoBio, Carlsbad, CA) was used to extract DNA from approximately 250 mg of feces after the metabolite extraction step in accordance with the manufacturer’s protocol with the following modifications: Bead beating was done at 6.5 m/s for 2 min using FastPrep-24 (M.P. Biomedicals, Irvine, CA), and tubes were centrifuged at 10k RCF for 1 min. Extracted DNA was stored at −80 °C until further analysis. The V4 region of the 16 S rRNA gene was amplified with the 515 F/806 R primer pairs with an 8 bp Hamming error-correcting barcode at the 3’ end of forward primer for sample multiplexing in later analysis. PCR was done with the following cycling parameters: 2 min of initial denaturation at 94 °C, followed by 25 cycles of denaturation for 45 sec at 94 °C, annealing for 60 sec at 50 °C, extension for 90 sec at 72 °C, with a final extension at 10 min at 72 °C. The integrity of the PCR products was confirmed by agarose gel electrophoresis and NanoDrop 2000c spectrophotometer (Thermo Scientific, Waltham, MA). The PCR amplicons were pooled and purified using QIAquick PCR Purification Kit (QIAGEN, Hilden, Germany) following the manufacturer’s protocol, which was then submitted to the UC Davis Genome Center DNA Technologies Core for 250 bp paired-end sequencing on Illumina Miseq.

### Sequencing data processing

Sequencing resulted in a total of 14.4 M reads, which were analyzed in Quantitative Insights Into Microbial Ecology (QIIME) pipeline version 1.8.0. Briefly, sequences were generated by merging the forward and reverse reads, and were demultiplexed. Chimeric sequences were identified and removed using the userch61 method. 8 samples were removed due to low sequencing reads (two control, one 1990s, and two 2008 at Infant; one 1990s and two 2008 at Juvenile time points). Sequences with 97% similarity were clustered into operational taxonomic units (OTUs) with the open-reference OTU picking method against the most recent Greengenes library (“gg_13_8_otus”). OTU counts that were less than 0.00005% of the total counts were removed from the OTU table^34^.

### Statistical analysis

Throughout the study, p-values from statistical analyses were adjusted using the Benjamini-Hochberg FDR procedure, and statistical significance was assumed only when adjusted p-values were less than 0.05. Metabolites and microbes with uncorrected p-values of less than 0.05 were also reported since FDR correction can increase the risk of false negative conclusions. In order to report the magnitude of difference between the groups as well as to consider the relatively small sample size, effect size and 95% confidence intervals were also calculated. All statistical analyses were done using R (version 3.3.2).

For metabolomics data analysis, the sample sizes for control, 1990s, and 2008 were 15, 7, and 9 for the Infant, and 14, 10, and 12 for the Juvenile time points respectively. Metabolite concentrations were log transformed, and the result was visualized using NMDS plots using the vegan (version 2.4-2) package of the R library (version 1.0.4) and the ggplot2 library (version 2.2.1). KW followed by the Mann-Whitney U test were applied in order to compare metabolomics data among groups at each time point due to the lack of normality of the data as well as the small sample size. In order to avoid the inflation of false positive rates in the Mann-Whitney U test, the Bonferroni method was used to obtain a new threshold^35^; and significance was only assumed when p-values were less than 0.05/3 = 0.017. Effect size (r) was calculated only for post-hoc tests since the effect size measurement for the KW test summarizes the general effects among the three groups, which is difficult to interpret^35^. Z values from the Mann-Whitney U test were calculated using the coin library (version 1.2.2) which was subsequently divided by the square-root of the total sample size for the post hoc test. The effect size is defined as follows: small (r < 0.3), medium (0.3 < r < 0.5), and large (0.5 < r)^35^. For microbiota analysis, the sample sizes for control, 1990s, and 2008 groups were 13, 7, and 7 for Infant, and 14, 9, and 10 for Juvenile time points respectively. OTU counts were rarefied at the minimum read counts (11162 reads), which was used for alpha and beta diversity analyses computed by phyloseq (version 1.19.1) in the R package. Beta diversity analysis was visualized by NMDS plots based on the weighted UniFrac distance. DESeq2 (version 1.14.1) was used to identify bacteria with statistically significant differences in the abundance among groups as DESeq2 has relatively high sensitivity and low false discovery rate with small datasets (<20 samples per group)^36^. P-values were calculated from the likelihood ratio test in DESeq2, and log2FoldChange was used to estimate the effect size. Bacterial abundance with estimated fold change of higher than 4 or lower than ¼ together with base mean of higher than 30 and adjusted p-value of less than 0.05 were considered to be significantly different^37^.

PERMANOVA was applied to compare the centroid of the three groups in the NMDS plots by utilizing the adonis function with the Bray-Curtis dissimilarity method in the R package of vegan (version 2.4.4), as well as the permutest to test the homogeneity of the group dispersion by using the betadisper function of the vegan package in R, followed by the TukeyHSD test as a post hoc test. Furthermore, for metabolome data, the influence of potential confounding factors, such as animal age at sample collection and drug administration (only at the Juvenile time point), on group differences were tested using non-parametric ANCOVA (R package ‘sm’)^38^ with Akaike information criterion with a correction for finite sample size (AICc) method^39^. For microbiota data, the likelihood ratio test was applied with the potential confounding factors included in the model. The effect of mode of delivery (C section or vaginal) was tested using NMDS for both datasets due to the small number of infants that were born via Caesarean section (Supplementary Table S1). Nine samples (5 from the Infant and 4 from the Juvenile time points) were removed as outliers because they showed distinct profiles from the samples collected at the same time point (Supplementary Figs S11–S13). Outliers were not explained by mode of delivery or drug administration (Supplementary Tables S1 and S3).

Bootstrapping was utilized to calculate the statistical power and 95% CI for the KW test for metabolome analysis and the likelihood ratio test for microbiota analysis. The sample sizes tested for the power calculation were the lowest and highest sample size of the three groups. 95% CI was obtained by using the groupwiseMedian function in rcompanion (version 1.13.2) in the R package. To determine the probability distribution, the number of bootstraps was set to 10,000 for the metabolome data. For microbiota data, 1,000 and 10,000 bootstraps were used for the statistical power calculation and 95% CI, respectively.

## Electronic supplementary material

Supplementary Material

## Data Availability

Accession codes for microbiota data is ERP107267.
