# Supplementary material for: Microbial structure and function in infant and juvenile rhesus macaques are primarily affected by age, not vaccination status
Source: Sci Rep. 2018 Oct 26;8:15867. doi: 10.1038/s41598-018-34019-0 (PMC6203732; doi:10.1038/s41598-018-34019-0)
Supplement: Supplementary file 1 — Supplementary Material [file 41598_2018_34019_MOESM1_ESM.pdf]

Microbial structure and function in infant and juvenile rhesus macaques are primarily affected by age, not vaccination status.

Yu Hasegawa[1], Britni Curtis[3], Vernon Yutuc[3], Megan Rulien[3], Kelly Morrisroe[3], Kristin Watkins[3], Clayton Ferrier[3], Chris English[3], Laura Hewitson[4*], and Carolyn M. Slupsky[1,2*]

[1] Department of Food Science and Technology, [2]Department of Nutrition, University of California, Davis, California, USA;

[3]Infant Primate Research Laboratory (IPRL), Washington National Primate Research Center, and Center on Human Development and Disability (CHDD), Seattle, Washington, USA;

[4]The Johnson Center for Child Health and Development, Austin, Texas, USA.

*Co-corresponding and co-senior authors:

Carolyn M. Slupsky	Laura Hewitson
Department of Nutrition	Research Director
University of California, Davis	The Johnson Center
cslupsky@ucdavis.edu	lhewitson@johnson-center.org
Tel: (530) 752-6804	(512) 732-8400

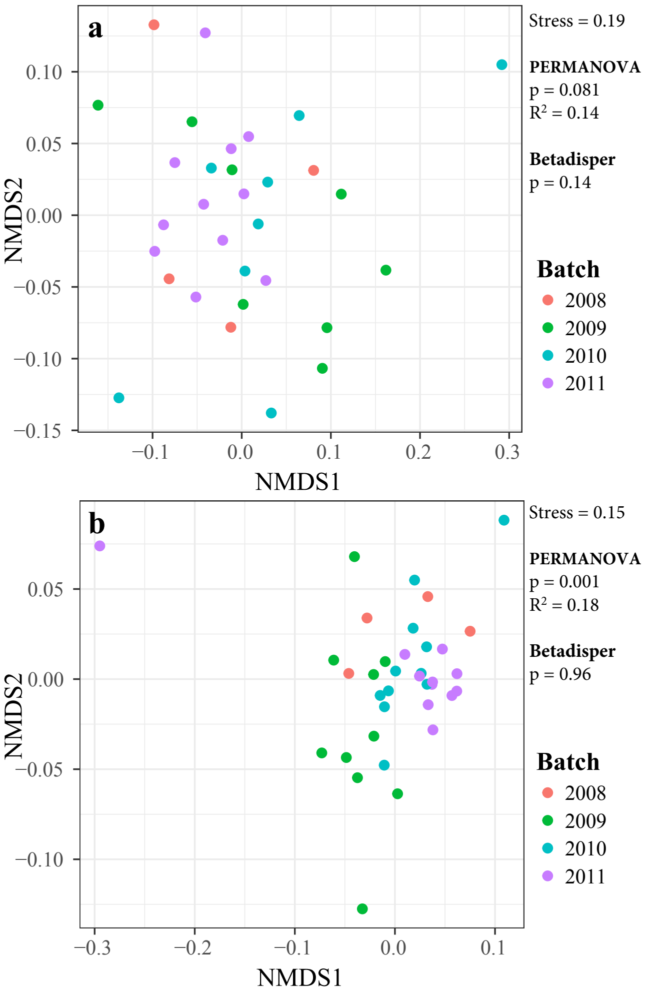

Supplementary Figure S1. NMDS plots showing the batch effect (birth year) on metabolome datasets at (a) Infant and (b) Juvenile time points. The stress value, as well as results from the PERMANOVA and betadisper analyses are included in each plot. 2008 (red), 2009 (green), 2010 (blue), and 2011 (purple).

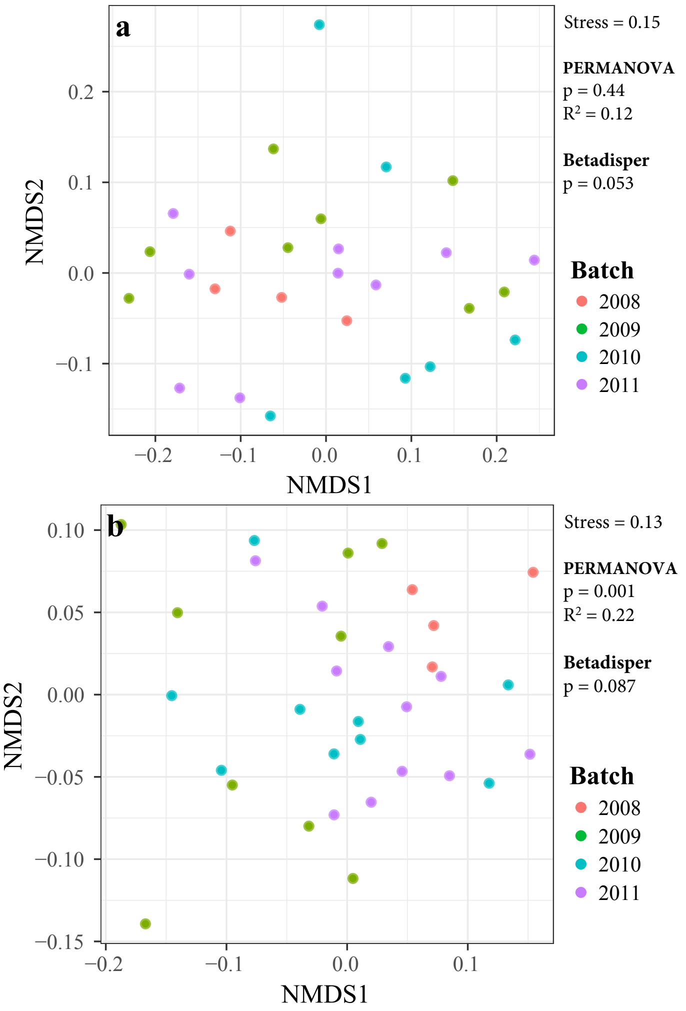

Supplementary Figure S2. NMDS plots showing the batch effect (birth year) on microbiota datasets at (a) Infant and (b) Juvenile time points. The stress value, as well as results from the PERMANOVA and betadisper analyses, are included in each plot. 2008 (red), 2009 (green), 2010 (blue), and 2011 (purple).

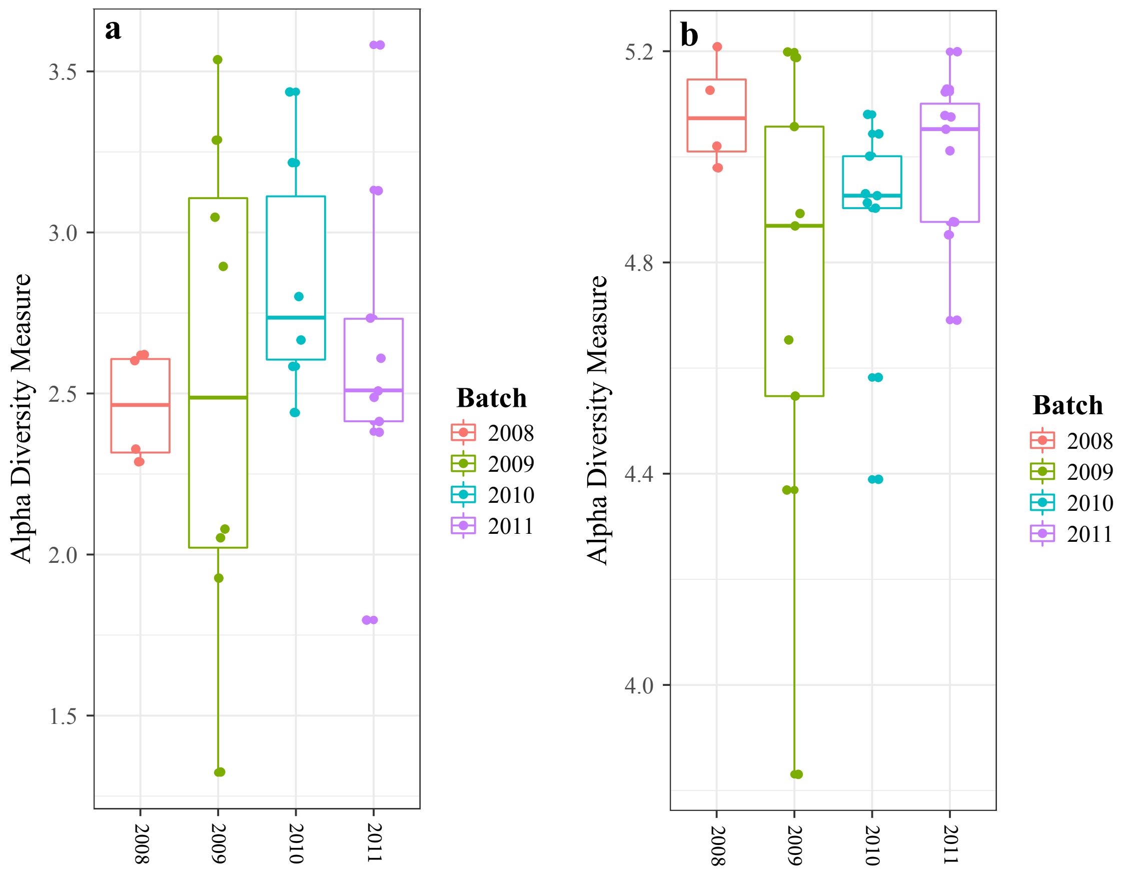

Supplementary Figure S3. Box plots for the alpha diversity values based on the Shannon method showing the batch effect (birth year) at the (a) Infant and (b) Juvenile time points. 2008 (red), 2009 (green), 2010 (blue), and 2011 (purple).

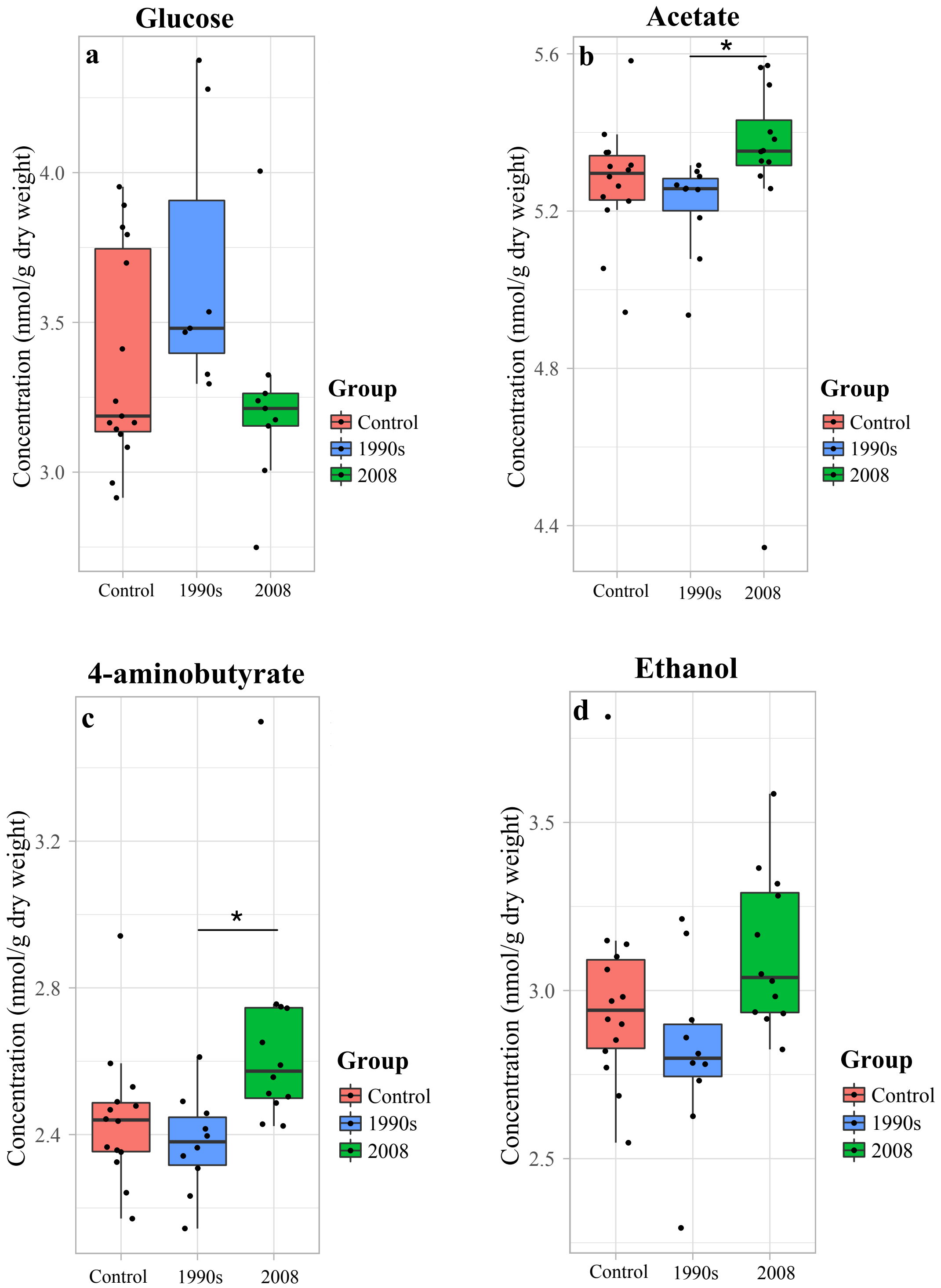
Glucose
a
Concentration (nmol/g dry weight)
4.0
3.5
3.0
Control
1990s
2008
Group
Control
1990s
2008
Acetate
b
5.6
5.2
4.8
4.4
*
Concentration (nmol/g dry weight)
Control
1990s
2008
Group
Control
1990s
2008
4-aminobutyrate
c
3.2
2.8
2.4
*
Concentration (nmol/g dry weight)
Control
1990s
2008
Group
Control
1990s
2008
Ethanol
d
3.5
3.0
2.5
Concentration (nmol/g dry weight)
Control
1990s
2008
Group
Control
1990s
2008

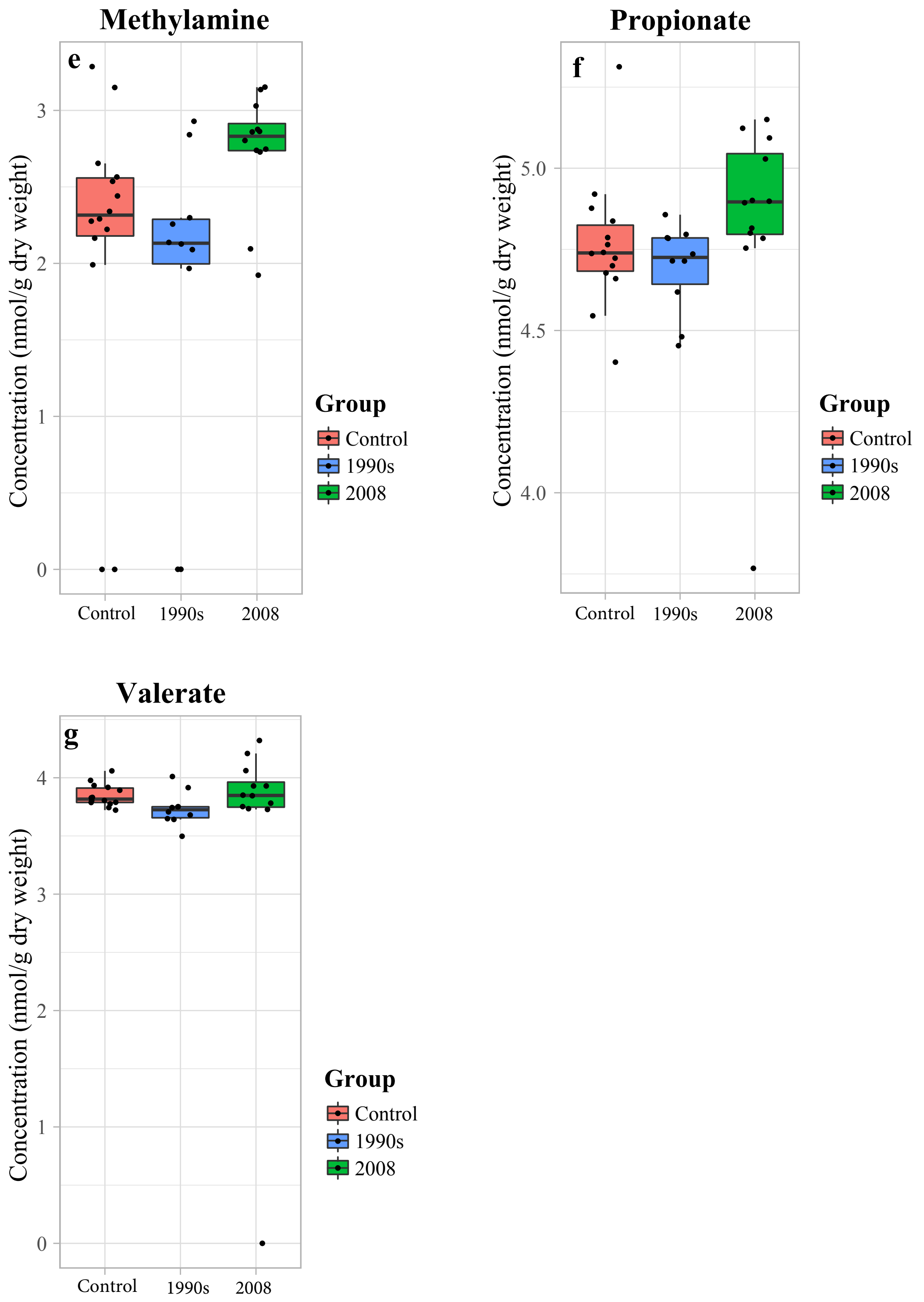

Supplementary Figure S4. Box plots showing the concentrations of metabolites with significant p-values by the KW test prior to the FDR correction. All plots were generated with the data collected at the Juvenile time point except for plot (a) for data collected at the Infant time point. Concentration of metabolites were log transformed. The Mann-Whitney U test was applied as a post hoc test. In order to avoid inflation of the false positive rate in the Mann-Whitney U test,

Bonferroni was used to obtain a new threshold, with significance assumed when p-values were less than 0.05/3=0.017. (*: $p<0.017$). Controls (red), 1990s (blue), and 2008 (green) groups.

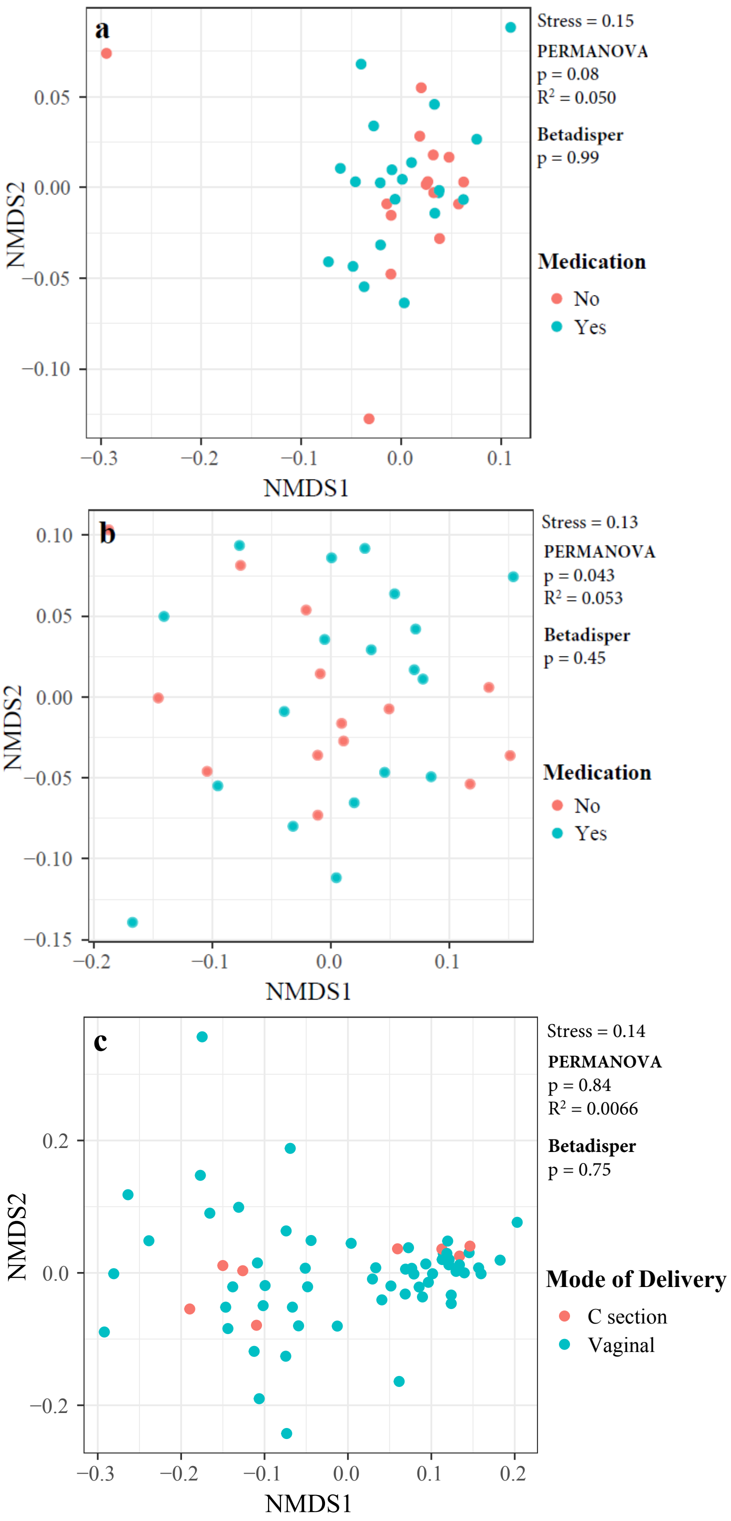
a
Stress = 0.15
PERMANOVA
p = 0.08
R² = 0.050
Betadisper
p = 0.99
Medication
No
Yes
NMDS1
NMDS2
b
Stress = 0.13
PERMANOVA
p = 0.043
R² = 0.053
Betadisper
p = 0.45
Medication
No
Yes
NMDS1
NMDS2
c
Stress = 0.14
PERMANOVA
p = 0.84
R² = 0.0066
Betadisper
p = 0.75
Mode of Delivery
C section
Vaginal
NMDS1
NMDS2

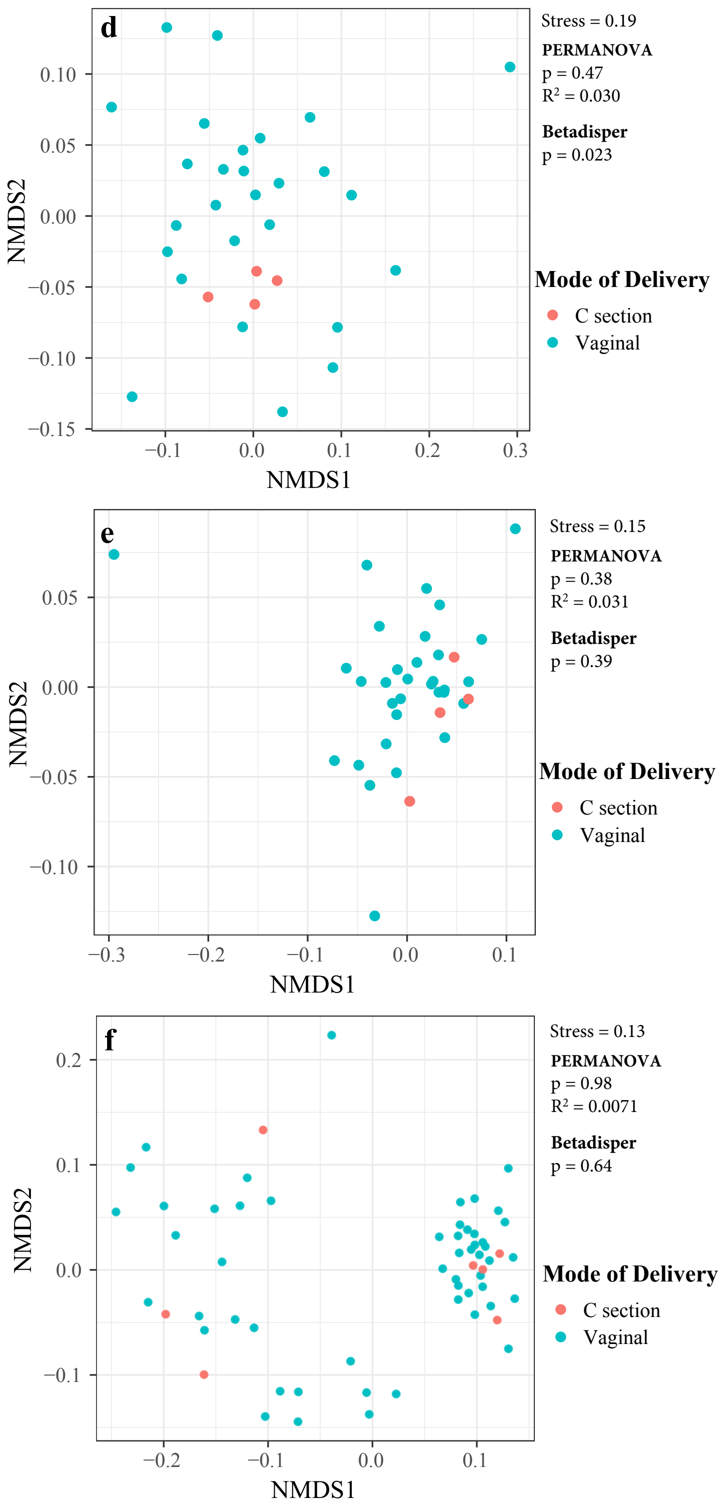
d
Stress = 0.19
PERMANOVA
p = 0.47
R² = 0.030
Betadisper
p = 0.023
Mode of Delivery
C section
Vaginal
NMDS2
NMDS1
e
Stress = 0.15
PERMANOVA
p = 0.38
R² = 0.031
Betadisper
p = 0.39
Mode of Delivery
C section
Vaginal
NMDS2
NMDS1
f
Stress = 0.13
PERMANOVA
p = 0.98
R² = 0.0071
Betadisper
p = 0.64
Mode of Delivery
C section
Vaginal
NMDS2
NMDS1

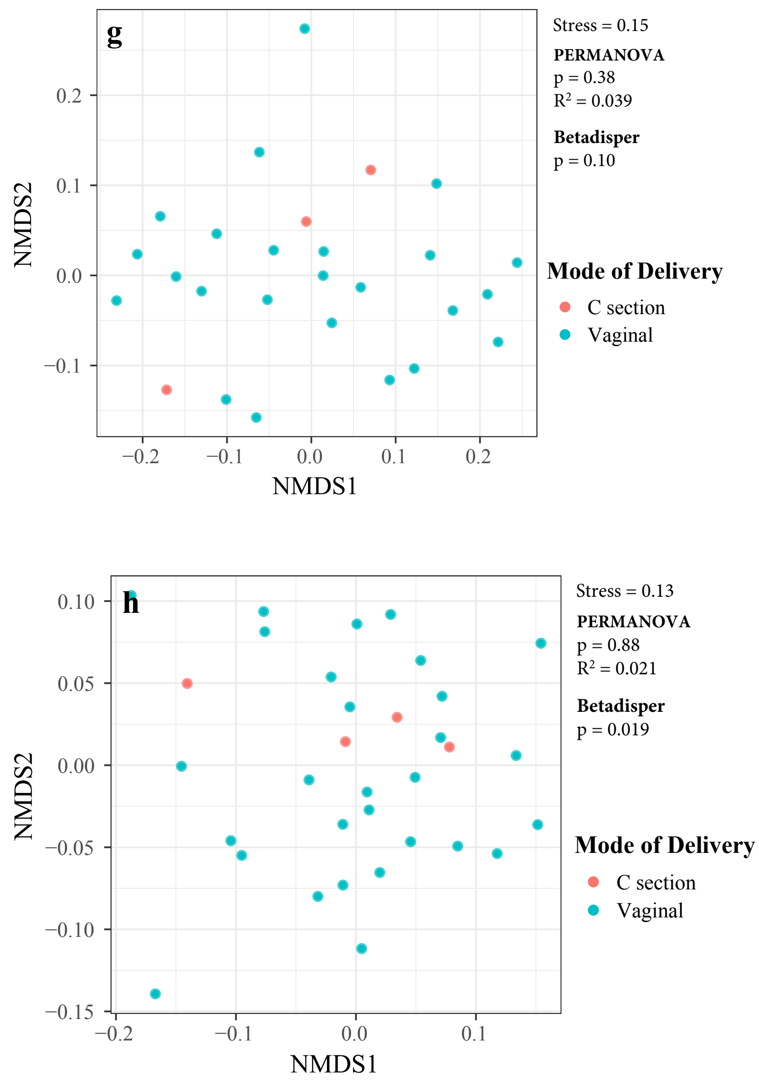

Supplementary Figure S5. The effect of potential confounding factors (medications or mode of birth) on metabolome and microbiota data. NMDS plots for analysis of medications for (a) metabolome, and (b) microbiota datasets at the Juvenile time point. NMDS plots for mode of birth for metabolome analysis (c) overall, (d) Infant, and (e) Juvenile time points, and for microbiota analysis (f) overall, (g) Infant, and (h) Juvenile time points. The stress value, as well as results from the PERMANOVA and betadisper analyses, are included in each plot.

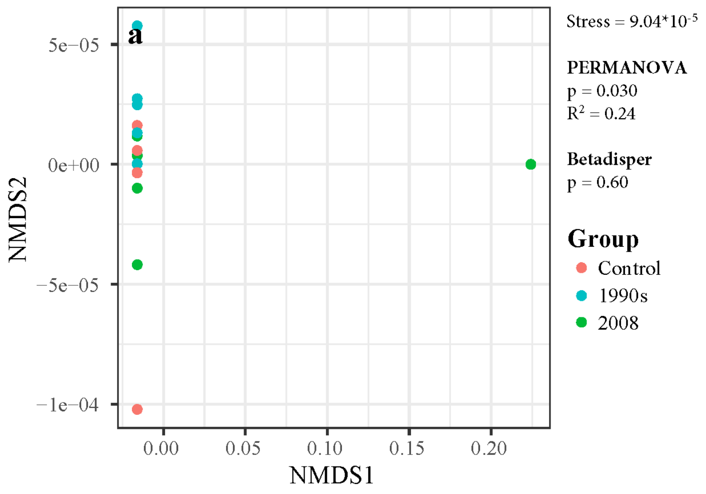

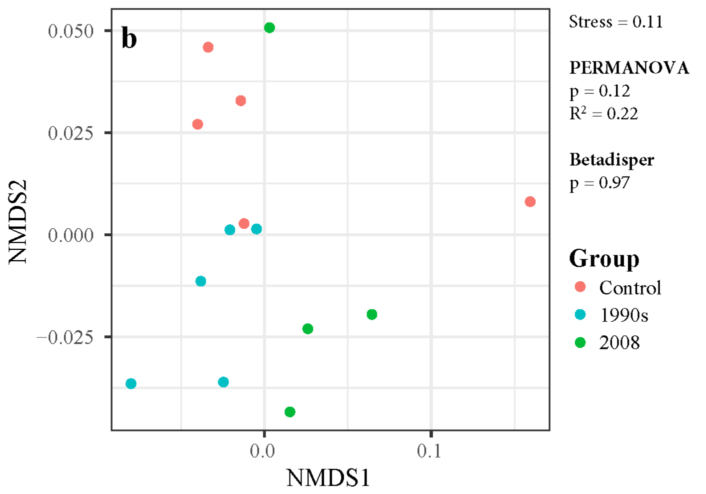

Supplementary Figure S6. NMDS plots on the metabolome dataset generated for animals reciving no medications. The outlier-like datapoint that made it difficult to observe the data distribution in plot (a) was removed to generate plot (b). (b) Although there seemed to be a cluster, PERMANOVA and betadisper tests did not show significant p-values (p=0.12 by PERMANOVA and p=0.97 by betadisper tests). Controls (red), 1990s (blue), and 2008 (green) groups.

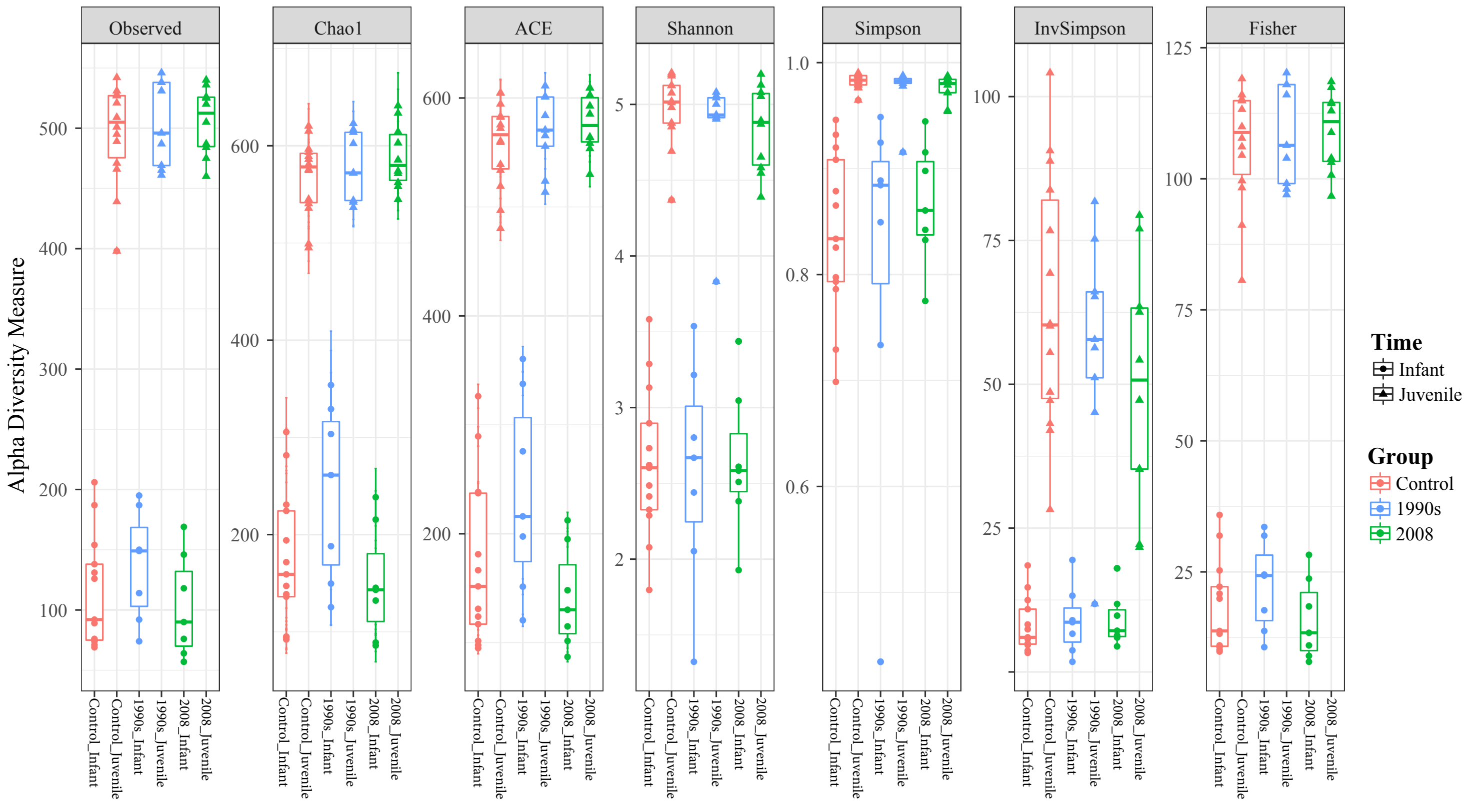

Supplementary Figure S7. Box plots showing the alpha diversity values based on the different methods. Circle represents data at Infant, triangle represents data at Juvenile time points. Controls (red), 1990s (blue), and 2008 (green) groups.

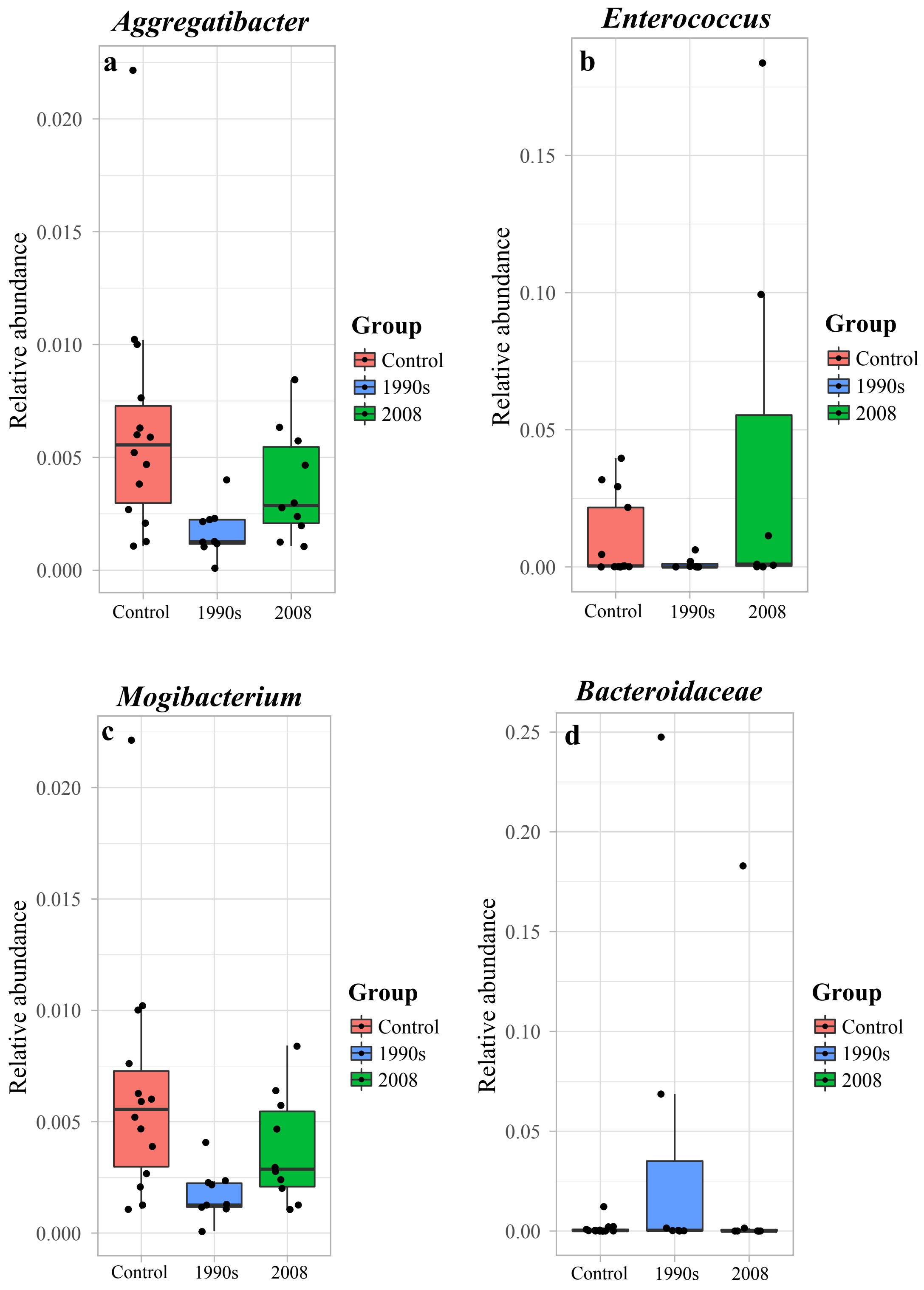

Aggregatibacter
a
Relative abundance
0.020
0.015
0.010
0.005
0.000
Control
1990s
2008
Group
Control
1990s
2008
Enterococcus
b
0.15
0.10
0.05
0.00
Mogibacterium
c
Bacteroidaceae
d
0.25
0.20

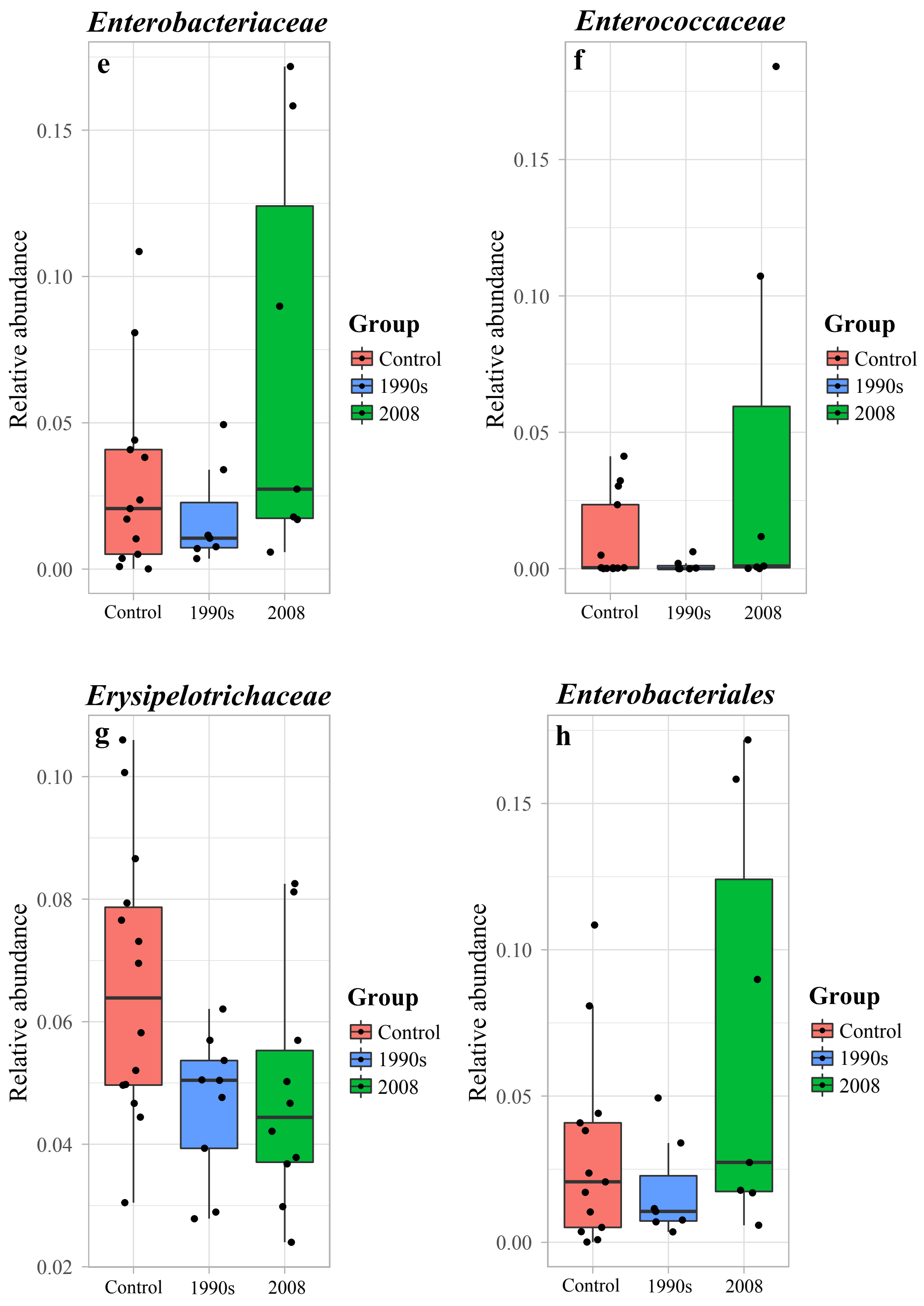
Enterobacteriaceae
e
Relative abundance
0.15
0.10
0.05
0.00
Control
1990s
2008
Group
Control
1990s
2008
Enterococcaceae
f
Relative abundance
0.15
0.10
0.05
0.00
Control
1990s
2008
Group
Control
1990s
2008
Erysipelotrichaceae
g
Relative abundance
0.10
0.08
0.06
0.04
0.02
Control
1990s
2008
Group
Control
1990s
2008
Enterobacteriales
h
Relative abundance
0.15
0.10
0.05
0.00
Control
1990s
2008
Group
Control
1990s
2008

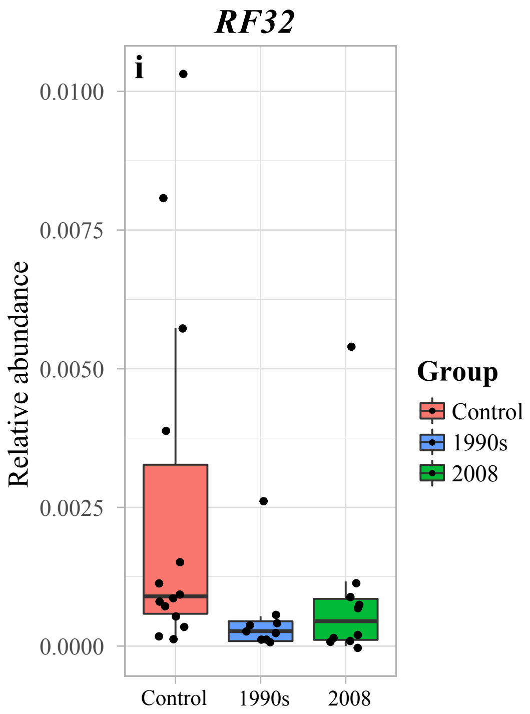

Supplementary Figure S8. Boxplots showing the relative abundance of bacteria with p-value of less than 0.05 prior to the FDR correction by the likelihood ratio test. At the genus level: (a) and (b) at the Infant and (c) at the Juvenile time point; at the family level: (d), (e) and (f) at the Infant and (g) at the Juvenile time point; at the order level: (h) at the Infant and (i) at the Juvenile time point. Controls (red), 1990s (blue), and 2008 (green) groups.

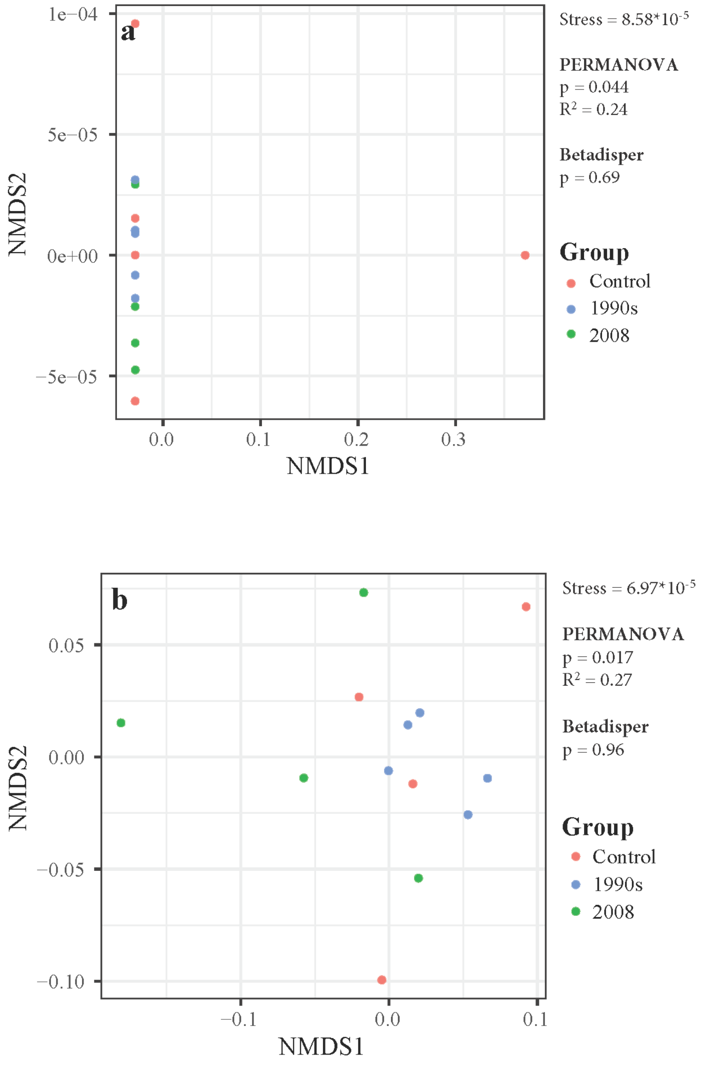

Supplementary Figure S9. NMDS plots on the microbiota dataset generated for animals receiving no medications (Juvenile time point). The outlier-like datapoint that made it difficult to observe the data distribution in plot (a) was removed to generate plot (b). (b) Although PERMANOVA test resulted in a significant p-values (p=0. 017), the R^2 value slows that relatively small portion of variation (27 %) could be explained by Group. Betadisper test showed insignificant p-value (p=0.96). Controls (red), 1990s (blue), and 2008 (green) groups.

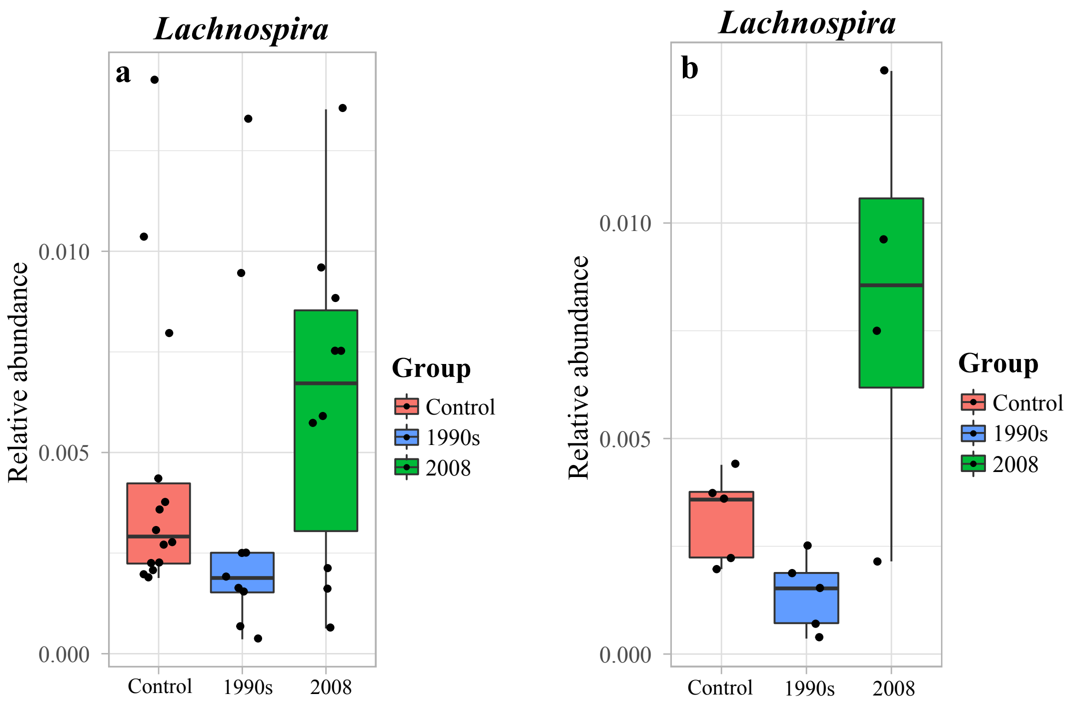

Supplementary Figure S10. Boxplots showing the relative abundance of *Lachnospira* (genus) with significant p-value with FDR correction by the likelihood ratio test. (a) was generated with all the animals available for the analysis, whereas (b) was generated for animals that did not receive any medications. Controls (red), 1990s (blue), and 2008 (green) groups.

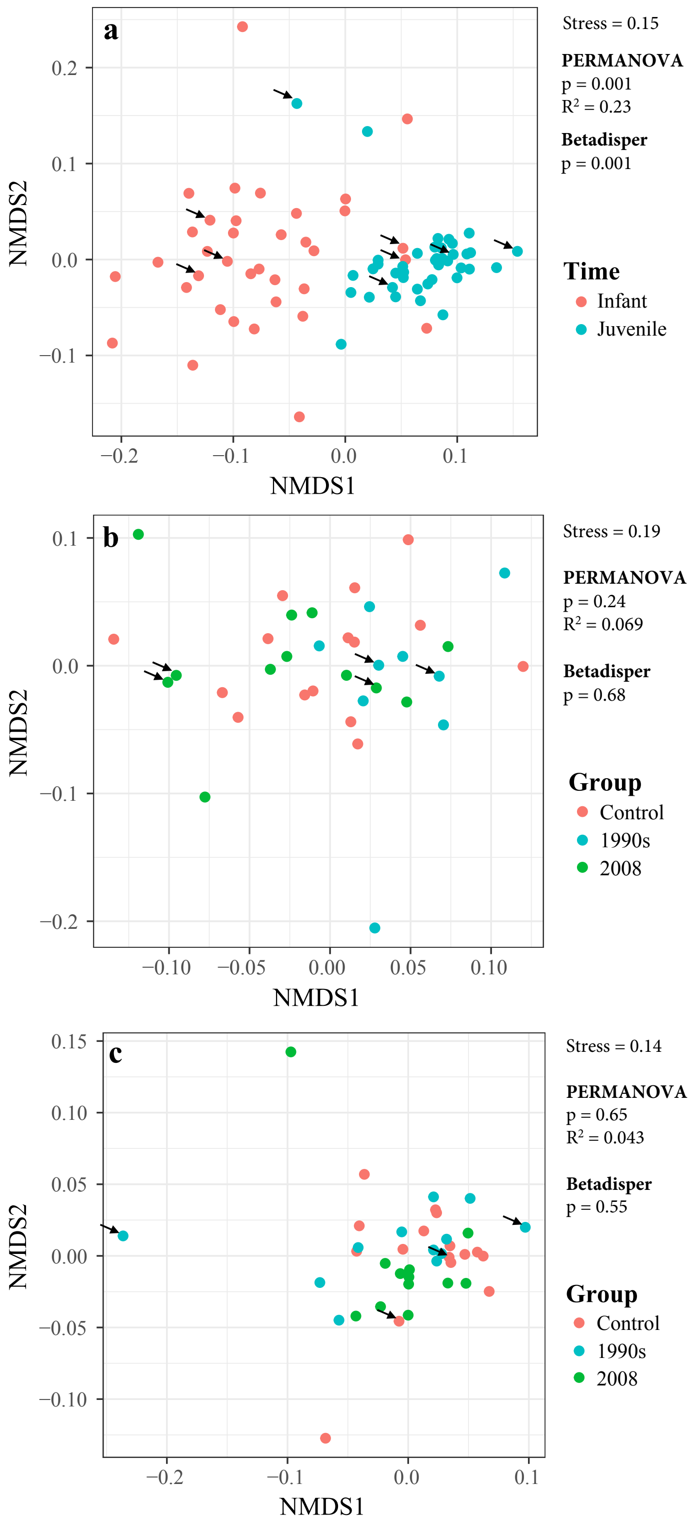

Supplementary Figure S11. NMDS plots for metabolomics analysis including outliers. (a) Overall profile of NMDS plots including data both at Infant (red) and Juvenile (blue) time points. NMDS plots for data collected from controls (red), 1990s (blue), and 2008 (green) at the (b) Infant and (c) Juvenile time points. Outliers are indicated with arrows.

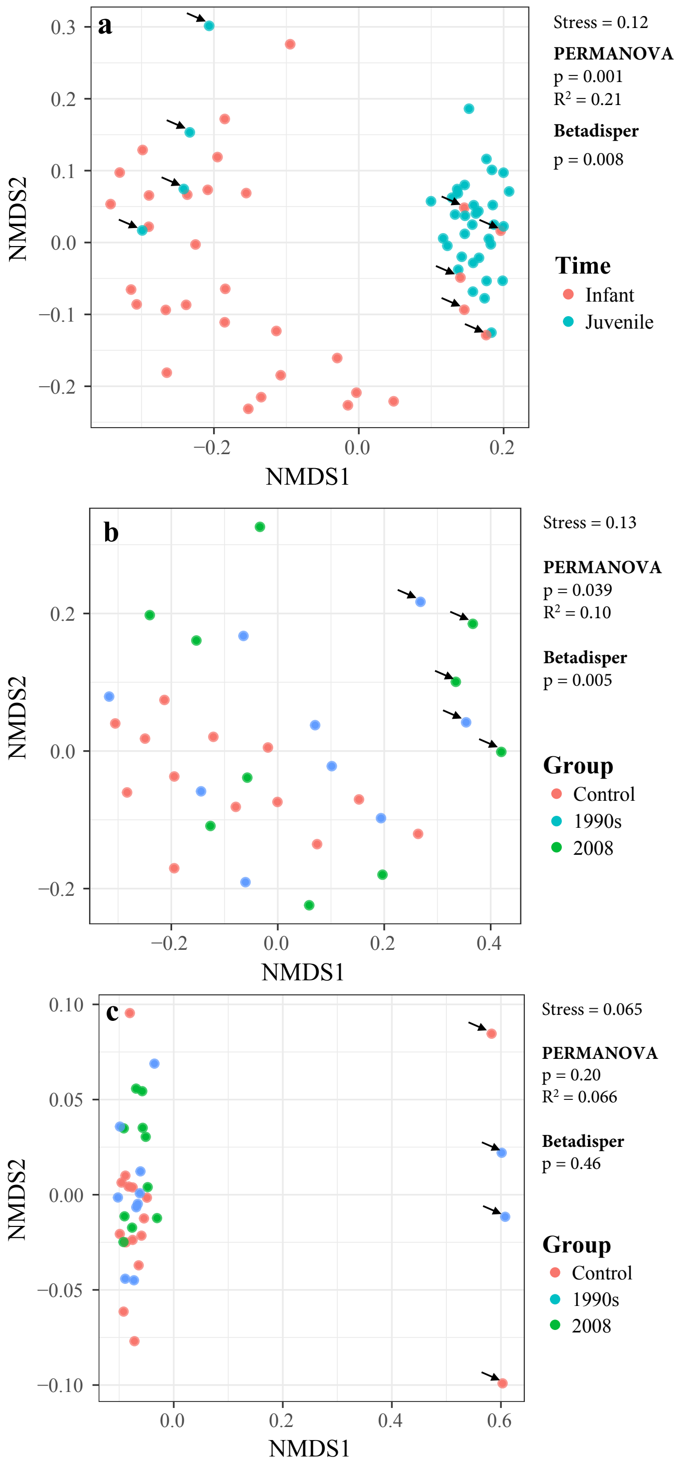

Supplementary Figure S12. NMDS plots for microbiota analysis including outliers, based on weighted UniFrac distance. (a) Overall NMDS plot including data from Infant (red) and Juvenile (blue) time points. NMDS plots for data collected from controls (red), 1990s (blue), and 2008 (green) groups at both (b) Infant and (c) Juvenile time points. Outliers are indicated with arrows.

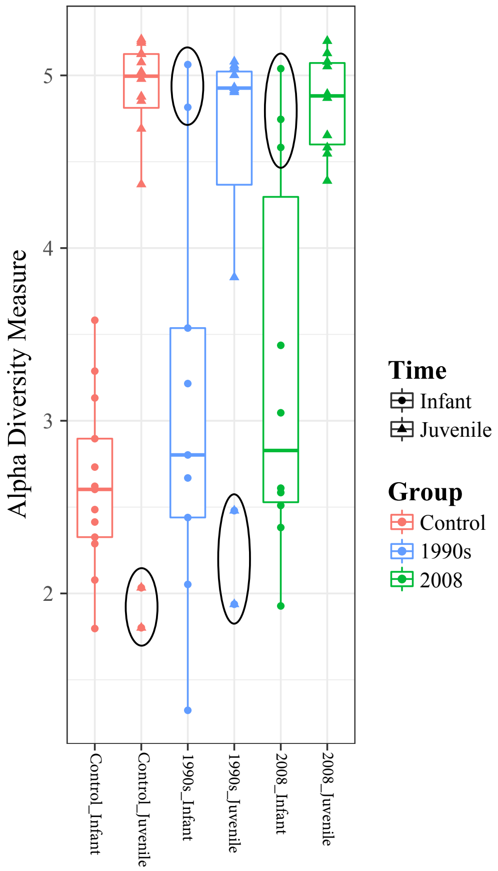

Supplementary Figure S13. Box plot showing the alpha diversity values based on Shannon analysis method including outliers. Circles represent data at the Infant time point, triangles represent data at the Juvenile time point. Outliers are indicated with circles. The middle line in the box represents median of the data, and the box represents lower and higher quantiles. The edges of wiskers represent the lowest and highest values of the dataset. Controls (red), 1990s (blue), and 2008 (green) groups.

Supplementary Table S1. Mode of delivery, year of birth, age at collection, sample availability, and study outliers for samples analyzed. Abbrev: N/A, sample not available.

Group	Animal ID	Mode of Delivery	Infant			Juvenile		
			Sample year	Animal age (days)	Outlier	Sample year	Animal age (days)	Outlier
Control	1	Vaginal	2008	5		2010	604	
	2	Vaginal	2008	7		2010	595	
	3	Vaginal	2008	9		2010	615	
	4	Vaginal	2008	7		2010	613	
	5	Vaginal	2009	8		2010	578	✓
	6	Vaginal	2009	7		2010	561	
	7	Vaginal	N/A	N/A		2010	559	
	8	Vaginal	2009	7		2010	548	
	9	Vaginal	2011	7		2012	571	
	10	Vaginal	2011	7		2012	564	
	11	Vaginal	2011	8		2012	563	✓
	12	Vaginal	2011	7		2012	549	
	13	C-section	2011	7		2013	563	
	14	Vaginal	2011	8		2013	563	
	15	Vaginal	2011	7		2013	551	
	16	Vaginal	2011	7		2013	543	
1990s	1	Vaginal	2009	9		2011	620	
	2	Vaginal	2009	7		2011	610	
	3	Vaginal	2009	6		2011	607	
	4	C-section	2009	8	✓	2011	595	✓
	5	Vaginal	N/A	N/A		2011	540	
	6	Vaginal	2010	7	✓	2011	538	
	7	Vaginal	2010	7		2011	537	
	8	Vaginal	2010	7		2011	530	
	9	C-section	2010	7		2012	563	✓
	10	Vaginal	2010	9		2012	557	
	11	Vaginal	2010	8		2012	556	
	12	Vaginal	N/A	N/A		2012	554	
2008	1	Vaginal	2009	7	✓	2010	562	
	2	Vaginal	2009	8		2010	554	
	3	Vaginal	2009	6		2010	545	
	4	C-section	2009	7		2010	543	
	5	Vaginal	2010	7	✓	2011	550	
	6	Vaginal	2010	7		2011	542	
	7	Vaginal	2010	6		2011	546	
	8	Vaginal	2010	7		2011	539	
	9	Vaginal	2011	7		2013	582	
	10	Vaginal	2011	7		2013	577	
	11	C-section	2011	7		2013	576	
	12	C-section	2011	7	✓	2013	576	

Supplementary Table S2. Summary of the result of the KW test on the metabolome dataset. Metabolites with significant p-values prior to FDR correction are listed. KW chi-squared value (χ^2) and statistical power, as well as the p-value and the effect size (r) of the Mann-Whitney U test as a post hoc test are included. From the effect size measurement, the magnitude of difference can be considered to be small when r<0.3, medium when 0.3<r<0.5, and large when 0.5<r. Median and 95% CI are also shown. In order to avoid inflation of the false positive rate in the Mann-Whitney U test, Bonferroni was used to obtain a new threshold, with significance assumed when p-values were less than 0.05/3=0.017.

Time point	KW test					Post-hoc test			Median; 95% CI [lower limit, upper limit]		
	**Metabolite**	**p-value**	**Adjusted p-value**	**KW chi-squared value**	**Estimated power (%)**	**Control vs 1990s**	**Control vs 2008**	**1990s vs 2008**	**Control**	**1990s**	**2008**
Infant	Glucose	0.049	0.91	6.02	66~97	p=0.23, r=0.38	p=1.00, r=0.042	p=0.024, r=0.67	3.19; [3.13, 3.79]	3.48; [3.33, 4.28]	3.21; [3.01, 3.32]
Juvenile	Acetate	0.013	0.27	8.62	75~89	p=0.62, r=0.26	p=0.20, r=0.36	**p=0.015, r=0.60**	5.30; [5.23, 5.35]	5.26; [5.17, 5.29]	5.35; [5.31, 5.46]
	4-aminobutyrate	0.0031	0.18	11.57	92~99	p=1.00, r=0.18	p=0.033, r=0.50	**p=0.0052, r=0.67**	2.44; [2.35, 2.49]	2.38; [2.29, 2.46]	2.57; [2.49, 2.75]
	Ethanol	0.019	0.27	8.06	73~88	p=0.69, r=0.25	p=0.28, r=0.33	p=0.019, r=0.59	2.94; [2.82, 3.10]	2.80; [2.70, 3.01]	3.04; [2.93, 3.30]
	Mythylamine	0.023	0.27	7.89	65~81	p=0.69, r=0.25	p=0.098, r=0.42	p=0.069, r=0.49	2.32; [2.17, 2.57]	2.13; [1.04, 2.55]	2.83; [2.73, 2.95]
	Propionate	0.018	0.27	7.57	70~86	p=1.00, r=0.13	p=0.11, r=0.41	p=0.023, r=0.58	4.74; [4.68, 4.83]	4.73; [4.60, 4.79]	4.90; [4.79, 5.06]
	Valerate	0.043	0.28	6.30	58~75	p=0.053, r=0.49	p=1.00, r=0.050	p=0.16, r=0.42	3.82; [3.79, 3.91]	3.73; [3.65, 3.83]	3.85; [3.74, 4.00]

Supplementary Table S3. Clinical indication for treatment by group, medications administered, length of treatment, and the number of days post treatment before juvenile stool samples were collected. *The number of days post treatment represents the length of time (days) that passed after treatment was completed until the juvenile stool sample was collected. For all but one animal (a control), at least 60 days passed. None of the animals had received any medications prior to the collection of the Infant sample.

Group	Animal ID	Clinical Indication	Medication	Length of Tx (days)	Number of days post Tx*
Control	1	Giardia - prophylactic	Metronidazole	14	83
	2	Giardia - prophylactic	Metronidazole	14	83
	3	Other bacterial infection	Amoxicillin clavulanate	6	542
	3	Giardia - prophylactic	Metronidazole	14	101
	4	Giardia - prophylactic	Metronidazole	14	101
	5	Giardia - prophylactic	Metronidazole	14	317
	6	Giardia - prophylactic	Metronidazole	14	317
	7	Other bacterial infection	Amoxicillin clavulanate	8	498
	7	Giardia - prophylactic	Metronidazole	14	318
	7	Diarrhea	Lactobacillus	13	498
	8	Giardia - prophylactic	Metronidazole	14	318
	8	Giardia	Metronidazole	14	337
	8	Diarrhea	Lactobacillus	5	346
	10	Other bacterial infection	Procaine penicillin G	4	25
	12	Other bacterial infection	Amoxicillin clavulanate	8	553
1990s	1	Giardia - prophylactic	Metronidazole	14	407
	2	Other bacterial infection	Enrofloxacin	10	377
	2	Giardia - prophylactic	Metronidazole	14	407
	3	Other bacterial infection	Amoxicillin clavulanate	14	538
	3	Giardia - prophylactic	Metronidazole	14	408
	4	Giardia - prophylactic	Metronidazole	14	408
	11	Giardia	Metronidazole	14	515
2008	1	Giardia - prophylactic	Metronidazole	14	254
	2	Giardia - prophylactic	Metronidazole	14	254
	3	Giardia - prophylactic	Metronidazole	14	255
	3	Diarrhea	Lactobacillus	7	479
	4	Other bacterial infection	Amoxicillin clavulanate	12	378
	4	Giardia - prophylactic	Metronidazole	14	255
	6	Other bacterial infection	Amoxicillin clavulanate	8	60
	9	Other bacterial infection	Amoxicillin clavulanate	8	511

Supplementary Table S4. The effect of potential confounding factors on the metabolome data analyzed by non-parametric ANCOVA. Age was included as the confounding factor for the Infant time point, whereas age and drug administration were added for the Juvenile time point. Metabolites that showed significant p-values prior to the FDR correction are included.

Time point	Metabolite	p-value	Adjusted p-value
Infant	Isovalerate	0.027	0.35
	Trimethylamine	0.036	0.35
Juvenile	4 Hydroxyphenylacetate	0.045	0.43
	Formate	0.036	0.43
	Fucose	0.025	0.43
	Taurine	0.039	0.43
	Uridine	0.017	0.43

Supplementary Table S5. Summary of the results of the KW test on the metabolome data for animals receiving no medications at the Juvenile time point (Sample size: control=5, 1990s=5, 2008=4). Metabolites with significant p-values prior to FDR correction are listed. Outliers found in Supplementary Figure S6 were removed. The KW chi-squared value (χ^2) and statistical power, as well as the p-value and the effect size (r) of the Mann-Whitney U test as a post hoc test are included. From the effect size measurement, the magnitude of difference can be considered to be small when $r<0.3$, medium when $0.3<r<0.5$, and large when $0.5<r$. Furthermore, median and the 95% CI are available. In order to avoid inflation of the false positive rate in the Mann-Whitney U test, Bonferroni was used to obtain the new threshold, with significance assumed when p-values were less than 0.05/3=0.017.

Time point	Metabolites	KW test				Post-hoc test			Median; 95% CI [lower limit, upper limit]		
		p-value	Adjusted p-value	KW χ2 value	Estimated power (%)	Control vs 1990s	Control vs 2008	1990s vs 2008	Control	1990s	2008
Juvenile	4_Aminobutyrate	0.030	0.29	7.05	71~93	p=0.18, r=0.63	p=1.00, r=0.16	p=0.060, r=0.82	2.47; [2.32, 2.94]	2.36; [2.14, 2.42]	2.55; [2.43, 2.75]
	Acetate	0.030	0.29	7.05	71~94	p=0.18, r=0.63	p=1.00, r=0.16	p=0.060, r=0.82	5.35; [5.23, 5.58]	5.25; [5.08, 5.30]	5.35; [5.33, 5.57]
	Butyrate	0.027	0.29	7.24	75~96	p=0.18, r=0.63	p=1.00, r=0.24	p=0.060, r=0.82	4.50; [4.35, 4.88]	4.41; [4.15, 4.46]	4.57; [4.52, 4.85]
	Hypoxanthine	0.0065	0.29	10.06	100	p=0.18, r=0.63	p=0.060, r=0.82	p=0.060, r=0.82	3.11; [2.97, 3.15]	3.02; [2.89, 3.06]	3.32; [3.17, 3.30]
	Methylamine	0.034	0.29	6.76	53~80	p=0.037, r=0.83	p=1.00, r=0.16	p=0.33, r=0.57	2.54; [2.34, 3.29]	2.13; [0, 2.26]	2.80; [2.09, 3.15]
	Nicotinate	0.031	0.29	6.94	74~94	p=0.28, r=0.56	p=1.00, r=0.33	p=0.060, r=0.82	2.83; [2.72, 2.95]	2.74; [2.40, 2.82]	2.90; [2.86, 3.06]
	Propylene glycol	0.022	0.29	7.60	85~98	p=0.28, r=0.56	p=0.054, r=0.83	p=0.68, r=0.45	2.01; [1.90, 2.87]	1.84; [0, 2.03]	0; [0, 1.54]

Supplementary Table S6. The batch effect on the metabolome data analyzed by non-parametric ANCOVA. Metabolites that showed significant p-values prior to the FDR correction at the Juvenile time point are included. At the Infant time point, no metabolites showed significant p-value even prior to FDR correction (data not shown).

Compound	p-value	Adjusted p-value
4-Aminobutyrate	0.029	0.14
Aspartate	0.039	0.14
Formate	0.027	0.14
Glutamate	0.030	0.14
Glycine	0.040	0.14
Hypoxanthine	0.036	0.14
Isoleucine	0.023	0.14
Isopropanol	0.034	0.14
Leucine	0.029	0.14
Methionine	0.047	0.15
myo-Inositol	0.050	0.15
Phenylalanine	0.027	0.14
Ribose	0.035	0.14
Serine	0.023	0.14
Succinate	0.030	0.14
Tyrosine	0.031	0.14
Uracil	0.031	0.14
Valerate	0.026	0.14
Valine	0.048	0.15
Xylose	0.032	0.14

Supplementary Table S7. The results of the KW test on the different alpha diversity measurement methods on the microbiota data at both time points.

**Statistical Test**	**Infant P value**	**Juvenile P value**
Chao1	0.88	0.63
ACE	0.40	0.54
Shannon	0.99	0.54
Simpson	0.82	0.39
InvSimpson	0.77	0.66
Fisher	0.62	0.15

Supplementary Table S8. List of bacteria that showed the highest average relative abundances in each group.

Time point	Rank	Infant		Juvenile	
		Name	Abundance (%)	Name	Abundance (%)
Control	1	*Bifidobacterium*	30.45	*Prevotella*	7.72
	2	*Blautia*	8.79	*Lactobacillus*	7.27
	3	*[Eubacterium]*	8.63	*Ruminococcus*	7.17
	4	*Catenibacterium*	6.57	*Blautia*	3.64
	5	*Collinsella*	5.92	*Coprococcus*	3.19
	6	*Streptococcus*	5.17	*[Eubacterium]*	2.80
	7	*Prevotella*	3.17	*Catenibacterium*	2.18
	8	*Lactobacillus*	1.92	*Dialister*	1.94
	9	*Bulleidia*	1.56	*Clostridium*	1.47
	10	*Dorea*	1.53	*Treponema*	1.44
1990s	1	*Bifidobacterium*	32.11	*Lactobacillus*	14.27
	2	*[Eubacterium]*	11.60	*Prevotella*	7.41
	3	*Prevotella*	7.04	*Ruminococcus*	6.35
	4	*Catenibacterium*	5.78	*Coprococcus*	3.79
	5	*Blautia*	5.65	*Blautia*	3.02
	6	*Bacteroides*	4.55	*Treponema*	2.30
	7	*Streptococcus*	3.65	*Dialister*	2.26
	8	*[Prevotella]*	3.59	*[Eubacterium]*	1.80
	9	*[Ruminococcus]*	2.35	*Catenibacterium*	1.60
	10	*Lactobacillus*	2.10	*Faecalibacterium*	1.40
2008	1	*Bifidobacterium*	20.10	*Lactobacillus*	14.04
	2	*[Eubacterium]*	11.30	*Prevotella*	9.34
	3	*Blautia*	8.99	*Ruminococcus*	4.34
	4	*Catenibacterium*	5.53	*Blautia*	4.12
	5	*Enterococcus*	4.23	*Coprococcus*	3.43
	6	*Lactobacillus*	3.75	*Dialister*	2.58
	7	*Streptococcus*	3.19	*[Eubacterium]*	1.90
	8	*Bacteroides*	2.63	*Faecalibacterium*	1.85
	9	*Clostridium*	2.36	*Treponema*	1.74
	10	*[Ruminococcus]*	2.33	*Dorea*	1.36

Supplementary Table S9. Summary of the results of likelihood ratio test on the bacterial abundance at the genus level. Bacteria with p-values less than 0.05 prior to FDR correction are included. The average of the normalized count values (baseMean), p-value, adjusted p-value, and the estimated power (%) are included. Also, the estimated fold difference in the bacterial abundance between the two group (log2FoldChange) is listed as the effect size measurement, as well as median and the 95% CI.

Time point	Bacteria taxonomy	baseMean	p-value	Adjusted p-value	Estimated power (%)	log2FoldChange			Median; 95% CI [lower limit, upper limit]		
						Control/1990s	Control/2008	1990s/2008	Control	1990s	2008
Infant	*Aggregatibacter*	60.07	0.047	0.80	41~46	0.97	-3.13	-4.10	1; [0, 25]	3; [0, 14]	18; [2, 308]
	Enterococcus	202.44	0.0034	0.12	48~52	3.27	-3.59	-6.85	4; [1, 242]	0; [0, 22]	11; [1, 1110]
Juvenile	*Mogibacterium*	46.60	0.0023	0.10	92~99	1.72	0.87	-0.85	62; [30, 85]	14; [12, 26]	32; [21, 64]

Supplementary Table S10. Summary of the results of likelihood ratio test on the bacterial abundance at the family level. Bacteria with p-values less than 0.05 prior to the FDR correction are included. The average of the normalized count values (baseMean), p-value, adjusted p-value, and the estimated power (%) are included. Also, the estimated fold difference in the bacterial abundance between the two group (log2FoldChange) is listed as the effect size measurement, as well as median and the 95% CI are available.

Time point	Bacteria taxonomy	baseMean	p-value	Adjusted p-value	Estimated power (%)	log2FoldChange			Median; 95% CI [lower limit, upper limit]		
						Control/ 1990s	Control/ 2008	1990s/2008	Control	1990s	2008
Infant	*Bacteroidaceae*	325.01	0.027	0.41	42~47	-5.61	-6.18	-0.56	2; [0, 9]	4; [1, 766]	0; [0, 16]
	Enterobacteriaceae	236.38	0.046	0.41	59~82	0.16	-1.45	-1.61	231; [57, 456]	118; [78, 379]	305; [189, 1770]
	Enterococcaceae	121.44	0.046	0.41	54~72	1.08	-2.94	-4.02	4; [1, 262]	0; [0, 22]	11; [1, 1200]
Juvenile	*Erysipelotrichaceae*	577.11	0.037	0.86	51~67	0.39	0.46	0.072	713; [554, 886]	563; [323, 636]	496; [378, 734]

Supplementary Table S11. Summary of the results of likelihood ratio test on the bacterial abundance at order level. Bacteria with p-values less than 0.05 prior to the FDR correction are included. The average of the normalized count values (baseMean), p-value, adjusted p-value, and the estimated power (%) are included. Also, the estimated fold difference in the bacterial abundance between the two group (log2FoldChange) is listed as the effect size measurement, as well as median and the 95% CI.

Time point	Bacteria taxonomy	baseMean	p-value	Adjusted p-value	Estimated power (%)	log2FoldChange			Median; 95% CI [lower limit, upper limit]		
						Control/1990s	Control/2008	1990s/2008	Control	1990s	2008
Infant	*Enterobacteriales*	325.59	0.035	0.58	66~84	0.39	-1.65	-2.04	231; [57, 456]	118; [78, 379]	305; [189, 1770]
Juvenile	*RF32*	15.76	0.031	0.56	63~80	2.11	1.20	-0.91	10; [6, 41]	3; [1, 6]	5; [1, 11]

Supplementary Table S12. The effect of potential confounding factors (age and/or administration of medications) on the microbiota data at the genus level was analyzed by the likelihood ratio test in DESeq2. Bacteria with p-values less than 0.05 prior to the FDR correction are included. The average of the normalized count values (baseMean), p-value, adjusted p-value, and log2FoldChange for each pair of groups are included.

**Time point**	**Bacteria taxonomy**	**baseMean**	**p-value**	**Adjusted p-value**	**log2FoldChange**		
					**Control/1990s**	**Control/2008**	**1990s/2008**
Infant	*Enterococcus*	202.44	0.0013	0.045	3.27	-3.59	-6.85
Juvenile	*Catenibacterium*	179.05	0.015	0.23	0.31	1.01	0.70
	Clostridium	130.89	0.030	0.35	1.13	0.51	-0.62
	[Eubacterium]	236.53	0.0068	0.16	0.48	0.67	0.19
	Mogibacterium	46.60	0.0015	0.069	1.72	0.87	-0.85

Supplementary Table S13. The batch effect on the microbiota data at the genus level was analyzed by the likelihood ratio test in DESeq2. Bacteria with p-values less than 0.05 prior to the FDR correction are included. The average of the normalized count values (baseMean), p-value, adjusted p-value, and log2FoldChange for each pair of groups are included.

**Time point**	**Bacteria taxonomy**	**baseMean**	**p-value**	**Adjusted p-value**	**log2FoldChange**		
					**Control/1990s**	**Control/2008**	**1990s/2008**
Infant	*Aggregatibacter*	60.07	0.038	0.65	0.97	-3.13	-4.10
	Enterococcus	202.44	0.0015	0.053	3.27	-3.59	-6.85
Juvenile	*Catenibacterium*	179.05	0.0079	0.12	0.31	1.01	0.70
	Clostridium	130.89	0.032	0.30	1.13	0.51	-0.62
	[Eubacterium]	236.53	0.0040	0.092	0.48	0.67	0.19
	Mogibacterium	46.60	0.0026	0.092	1.72	0.87	-0.85

Supplementary Table S14. Likelihood ratio test in DESeq2 was applied to the animals receiving no medications at the Juvenile time point. The outlier found in Supplementary Figure S9 was removed in this analysis. No animals at the Infant time point had received any medications. Bacteria with p-values less than 0.05 prior to the FDR correction are included. The average of the normalized count values (baseMean), p-value, adjusted p-value, and log2FoldChange for each pair of groups are included.

**Time point**	**Bacteria taxonomy**	**baseMean**	**p-value**	**Adjusted p-value**	**log2FoldChange**		
					**Control/1990s**	**Control/2008**	**1990s/2008**
Juvenile	*Lachnospira*	43.71	0.00013	0.0058	1.23	-1.32	-2.56

Supplementary Table S15. Vaccine source, ethylmercury (EtHg) content for thimersoal-containing vaccines created specifically for the study, and route of vaccine administration. Abbr: IM, intramuscular; sub Q, subcutaneous. [a] These vaccines were thimerosal-free for the 2008 schedule. [b] For pregnant dams only. Modified with permission[11].

Vaccine	Trade name (Manufacture) NDC #	EtHg content (µg/0.5 ml dose)	Route of Administration
Hepatitis B	Recombivax HB (Merck) 0006-4981-00	1.98[a]	IM
Diphtheria, Tetanus, acellular Pertussis	Infanrix (GlaxoSmithKline) 58160-810-46	3.97[a]	IM
Haemophilus Influenza B	ActHIB (Sanofi Pasteur) 49281-545-05	3.96[a]	IM
Measles Mumps Rubella	MMR-II (Merck) 0006-4682-00	N/A	sub Q
Inactivated Polio vaccine	IPOL (Sanofi Pasteur) 49281-860-10	N/A	IM
Rotavirus	Rotateq (Merck) 0006-4047-41	N/A	Oral gavage
Pneumococcal 7- valent Conjugate Vaccine	Prevnar (Wyeth) 0005-1970-67	N/A	IM
Hepatitis A	VAQTA (Merck) 0006-4831-41	N/A	IM
Varicellar	Varivax (Merck) 0006-4827-00	N/A	sub Q
Meningococcal Polysaccharide Vaccine	Menomune (Sanofi Pasteur) 49281-489-05	3.96	sub Q
Influenza	Fluzone (Sanofi Pasteur) 49281-009-50	3.96	IM
Influenza	Fluzone (Sanofi Pasteur) 49281-382-15	25[b]	IM
